# Ginsenoside Rh1 Induces MCF-7 Cell Apoptosis and Autophagic Cell Death through ROS-Mediated Akt Signaling

**DOI:** 10.3390/cancers13081892

**Published:** 2021-04-15

**Authors:** Diem Thi Ngoc Huynh, Yujin Jin, Chang-Seon Myung, Kyung-Sun Heo

**Affiliations:** 1 College of Pharmacy, Chungnam National University, Daejeon 34134, Korea; ngocdiemphar@gmail.com (D.T.N.H.); 201850535@o.cnu.ac.kr (Y.J.); cm8r@cnu.ac.kr (C.-S.M.); 2 Department of Pharmacy, Da Nang University of Medical Technology and Pharmacy, Da Nang 550000, Vietnam

**Keywords:** Akt, apoptosis, autophagy, ginsenoside Rh1, reactive oxygen species

## Abstract

**Simple Summary:**

Breast cancer (BC) is the most common cause of cancer-related deaths among women worldwide, and its incidence has been increasing. However, current therapeutic approaches, such as chemotherapy, radiation, and hormonal therapy, have become increasingly ineffective because of their severe adverse effects and multidrug resistance. Therefore, the discovery of new potential candidates for BC therapy is essential. Here, we investigated whether ginsenoside Rh1 exhibits anticancer effects on BC. We found that this ginsenoside effectively inhibited the growth of BC cells in both cell cultures and mice. Therefore, ginsenoside Rh1 is a promising candidate for BC treatment.

**Abstract:**

Breast cancer (BC) is the leading cause of cancer-related deaths among women worldwide. Ginsenosides exhibit anticancer activity against various cancer cells. However, the effects of ginsenoside Rh1 on BC and the underlying mechanisms remain unknown. Here, we investigated the anticancer effects of Rh1 on human BC MCF-7 and HCC1428 cells and the underlying signaling pathways. The anticancer effects of Rh1 in vitro were evaluated using sulforhodamine B (SRB), 3-(4, 5-dimethylthiazole-2-yl)-2, 5-diphenyltetrazolium bromide (MTT), clonogenic assay, propidium iodide (PI)/Hoechst staining, Western blotting, flow cytometry, and immunofluorescence analysis. The in vivo effects of Rh1 were determined using a xenograft model via hematoxylin and eosin and the immunohistochemistry staining of tumor tissues. We found that Rh1 exerted cytotoxicity in the cells by increasing cell apoptosis, autophagy, and cell cycle arrest. These effects were further enhanced by a phosphatidylinositol 3-kinase (PI3K) inhibitor but were rescued by the inhibition of reactive oxygen species (ROS). Moreover, enhanced ROS generation by Rh1 inhibited the activation of the PI3K/Akt pathway. Consistently, Rh1 treatment significantly reduced tumor growth in vivo and increased the ROS production and protein expression of LC3B and cleaved caspase-3 but decreased the phosphorylation of Akt and retinoblastoma (Rb) in tumor tissues. Taken together, Rh1 exerted a potential anticancer effect on BC cells by inducing cell cycle arrest, apoptosis, and autophagy via inhibition of the ROS-mediated PI3K/Akt pathway.

## 1. Introduction

Cancer is a major global health issue that has been increasing dramatically. Among the different types of cancer, breast cancer (BC) is commonly associated with cancer-related deaths in women [1]. Chemotherapy, radiation, hormonal therapy, and surgery have been used as therapies for BC. However, because of severe adverse effects and multidrug resistance, these therapeutic approaches have become increasingly ineffective [2]. Therefore, discovering new potential candidates for BC therapy is essential, and natural compounds have been attracting great attention owing to their promising anticancer properties [3].

Reactive oxygen species (ROS), which are oxygen-containing reactive molecules, include superoxide anions, hydroxyl radicals, singlet oxygen, and hydrogen peroxide. The excessive production of ROS can promote several types of cell death, such as apoptosis, autophagy, ferroptosis, and necrosis [4]. Emerging data have demonstrated that elevated ROS production leads to cytotoxicity in cancer cells via different mechanisms, including cell cycle arrest, apoptosis, and autophagy promotion [4,5,6,7,8]. The deficiency of programmed cell death increases the life expectancy of neoplastic cells, which promotes cancer progression [9]. Apoptosis, a type of programmed cell death, is a common target of anticancer drugs [9,10]. Apoptosis is promoted via both intrinsic and extrinsic pathways [9]. These pathways lead to the activation of caspases, cysteine proteases that cleave different substrates, and, subsequently, cause cell death. Notably, numerous ginsenosides exert anticancer effects through different mechanisms, such as the induction of apoptosis and autophagy, by increasing ROS generation [5,6,7]. 

Uncontrolled cell proliferation in cancer is attributed to the dysregulation of cell cycle progression; therefore, cell cycle regulation is considered a potential target in cancer therapy [11]. There are four distinct phases of the cell cycle, including the G0/G1, S, G2, and M phases. Cyclin-dependent kinases (CDKs) play a critical role in the control of cell cycle progression. Recently, several CDK4/6 inhibitors, such as Palbociclib, ribociclib, and abemacicliz, have been accepted by the Food and Drug Administration (FDA) for the treatment of hormone receptor-positive and human epidermal growth factor receptor 2 (HER2)-negative BC [12]. Activated CDK4 and CDK6 promote cell cycle progression from the G0/G1 to S phase [11]. The activity of these CDKs is positively regulated by interactions with D-type cyclins (D1, D2, and D3) and negatively regulated by the binding to CDK inhibitors. In addition, cyclin D-CDK4/6 complexes drive cell cycle progression by sequestering p21^CIP1^ and p27^KIP1^, as well as by phosphorylating various targets, especially the retinoblastoma (Rb) tumor-suppressor protein [11]. p21^CIP1^ and p27^KIP1^ are two CDK inhibitors that bind to and prevent the activation of cyclin E-CDK2 kinase [11]. 

Moreover, later studies revealed that autophagy can act as a tumor suppressor and, therefore, can be a target for cancer therapy [13]. The autophagy process consists of five phases: initiation, nucleation, maturation, fusion, and degradation, and is regulated by various factors [14]. mTORC1 is a repressor of autophagic responses, and mTORC1 inhibition results in an increase in autophagy [14]. In addition, autophagy-related (ATG) proteins and their core complexes, such as Unc-51-like autophagy activating the kinase core complex, autophagy-specific class III phosphatidylinositol 3-kinase (PI3K) complex, ATG12, and LC3 ubiquitin-like conjugation systems, are associated with autophagy progression [13]. Excessive autophagy can lead to type II programmed cell death through the degradation of the mitochondria and molecules critical for cell survival [13]. 

The PI3K/Akt/mTOR signaling pathway is frequently stimulated in various human cancers, contributing to the regulation of various processes of cancer development, such as metabolism, proliferation, apoptosis, chemoresistance, and genomic instability [15,16]. The PI3K/Akt signaling pathway is known to play a crucial role in controlling the autophagy process [13]. On the other hand, this pathway contributes to the regulation of cell survival and enhanced protection of BC cells from apoptosis during tumorigenesis [17]. It has been demonstrated that PI3K inhibition leads to a decrease in cell growth and an increase in cell death in cancer cells [18]. Therefore, PI3K/Akt inhibition has been suggested as an important anticancer strategy [18]. 

Ginsenosides are the major bioactive compounds of the root of *Panax ginseng* Meyer, which display a variety of pharmacological activities, such as anti-inflammatory, anticancer, antidiabetic, etc. [5,19,20]. Ginsenosides are divided into three types based on aglycone moieties: protopanaxadiol-type ginsenosides, protopanaxatriol-type ginsenosides, and oleanolic acid-type ginsenosides [5]. Among them, ginsenoside Rh1 (Rh1) has been identified as a protopanaxatriol-type ginsenoside, which possesses anticancer activities in several cancer cell lines, such as human lung carcinoma, human cervix uterine adenocarcinoma, colorectal cancer cells, and malignant glioma cells [19,21,22]. Rh1 has been found to significantly induce cytotoxicity in human lung cancer cells from 100 µM [23], inhibit colorectal cancer cell proliferation from 50 µM [21], and suppress malignant glioma cell invasion at 300 µM [22]. However, the anticancer effect of Rh1 on BC cells and the underlying mechanisms remain to be elucidated. In this study, we investigated the anticancer effects of Rh1 on MCF-7 and HCC1428 BC cells and their potential mechanisms. 

## 2. Results

### 2.1. Rh1 Showed Anticancer Effects on BC Cells

**Figure 1:**
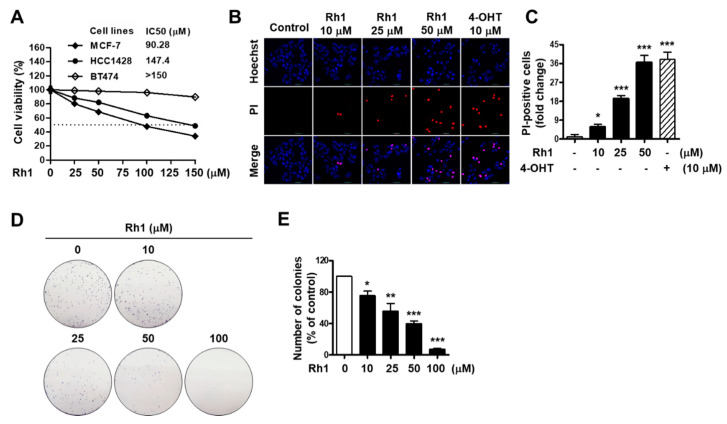
Anticancer effect of Rh1 on breast cancer (BC) cells. (**A**) MCF-7, HCC1428, and BT474 cells were treated with Rh1 (0, 25, 50, 100 and 150 µM) for 72 h. Cell viability was evaluated using the SRB assay. (**B**,**C**) MCF-7 cells were treated with Rh1 (10, 25, and 50 µM) or 4-OHT (10 µM) for 24 h. 4-OHT was used as a positive control. After the double staining with PI and Hoechst, the cells were observed under a fluorescent microscope, and the numbers of dead cells were quantified; a total of 5 fields per sample were counted. The cells stained with PI (red fluorescence) were indicated as dead cells. Images are shown at ×200 magnification. (**D**,**E**) Colony formation was assayed on a 6-well plate for 10 days, and then, the cells were fixed with 4% formaldehyde and stained with 0.5% crystal violet. Representative images, which are shown at ×1 magnification (**E**), and quantified bar graph (**F**) indicate the colony formation of MCF-7 cells. (**A**,**C**,**E**) Data are expressed as the mean ± SD (*n = 3*), * p < 0.05, ** p < 0.01, and *** p < 0.001 as compared with the control. 4-OHT: 4-hydroxytamoxifen, SRB: sulforhodamine B, PI: propidium iodide, and IC_50_: half-maximal inhibitory concentration.

To investigate the anticancer effects of Rh1 on BC, MCF-7, HCC1428, and BT474 cells were treated with different concentrations of Rh1 (25, 50, 100, and 150 µM) for 72 h, and a sulforhodamine B (SRB) assay was then performed to analyze the cell viability. Four-parameter logistic regression was also assessed to determine the half-maximal inhibitory concentration (IC_50_) values. The IC_50_ values of Rh1 in MCF-7, HCC1428, and BT474 cells were 90.28 µM, 147.4 µM, and >150 µM, respectively. The highest inhibitory effect of Rh1 on the cell viability was observed in the MCF-7 cells (Figure 1A). Next, to confirm the effects of Rh1 on the cell viability with morphological changes, MCF-7 cells were stained with the red fluorescent dye propidium iodide (PI) and counterstained with Hoechst 33342 nucleic acid stain for nuclei indication. PI-stained dead cell nuclei was significantly induced by Rh1 treatment; particularly, 50-µM Rh1-induced dead cell nuclei populations were similar to 10-µM 4-hydroxytamoxifen (4-OHT) treatment (Figure 1B,C). Furthermore, the inhibitory effect of Rh1 was confirmed using a clonogenic assay. Colony formation was significantly decreased by treatment with Rh1 from a concentration of 10 µM; in particular, Rh1 almost suppressed the cell reproductive viability at a concentration of 100 µM (Figure 1D,E). 

### 2.2. Rh1 Induced Cell Apoptosis and Cell Cycle Arrest in BC Cells

**Figure 2:**
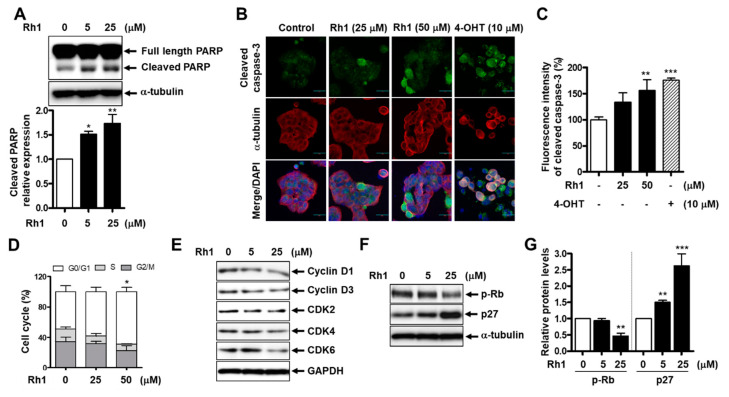
Rh1 induced cell apoptosis and cell cycle arrest in MCF-7 cells. (**A**) The cells were treated with Rh1 (0, 5, and 25 µM) for 24 h. Whole-cell lysates were then used to analyze the protein expression as indicated. α-tubulin was used as a loading control. (**B**,**C**) The cells were treated with Rh1 (0, 25, and 50 µM) or 4-OHT (10 µM) for 24 h. An immunofluorescence analysis was performed after fixing and immunostaining. Images are shown at ×200 magnification (**B**). Scale bar indicates 30 μm. Quantified graph showed the relative fluorescent intensity of cleaved caspase-3 using Image J software (**C**). (**D**) MCF-7 cells were treated with Rh1 at different concentrations for 24h. After the treatment, the cells were harvested for the cell cycle distribution analysis using flow cytometry. (**E**) The cells were treated with Rh1 (0, 5, and 25 µM) for 24 h. Total cell lysates were analyzed by Western blotting using the indicated antibodies. (**F**,**G**) The cells were treated with Rh1 (0, 5, and 25 µM) for 24 h. Total cell lysates were analyzed by Western blotting using the indicated antibodies (**F**). The relative quantification of the protein levels was analyzed by Image J software (**G**). (**A**,**C**,**D**,**G**) Data are expressed as the mean ± SD (*n = 5*), * p < 0.05, ** p < 0.01, and *** p < 0.001 as compared with the control. 4-OHT: 4-hydroxytamoxifen, CDK: cyclin-dependent kinase, and GAPDH: glyceraldehyde 3-phosphate dehydrogenase.

Next, we investigated whether Rh1 mediates the apoptotic pathway in BC cells. MCF-7 cells were pretreated with 10-µM Z-VAD-FMK, a caspase inhibitor, before treatment with Rh1 for 24 h and were subjected to the SRB assay. Rh1-decreased cell viability was partially recovered by Z-VAD-FMK, indicating that Rh1-induced cell death is related to apoptosis (Figure S3). Next, Rh1 treatment strongly increased the expression of the apoptosis-related protein, cleaved PARP, from 5 µM in MCF-7 cells (Figure 2A) and in HCC1428 cells (Figure S1A). In addition, the 50-µM Rh1-treated group showed increased cleaved caspase-3 expression, similar to the 10-µM 4-OHT-treated group (Figure 2B,C). 4-OHT was used as a positive control for cleaved caspase-3 enhancement.

In addition, to identify whether Rh1-decreased cell viability is involved in cell cycle arrest, a flow cytometry analysis was used to detect the cell cycle distribution in MCF-7 cells treated with Rh1. Compared to the control, cells incubated with Rh1 (25 and 50 µM) for 24 h showed an increase in G0/1-phase accumulation (Figure 2D). Consistently, treatment with Rh1 reduced the expression of cell cycle-related proteins, including cyclin D1, cyclin D3, CDK2, CDK4, and CDK6 (Figure 2E). Additionally, Rh1 led to a significant reduction in phosphorylated Rb protein levels and a remarkable increase in the protein expression of p27 in MCF-7 cells (Figure 2F,G) and HCC1428 cells (Figure S1B). Taken together, these data suggest that Rh1 possesses anticancer effects in MCF-7 cells by promoting cell cycle arrest and the apoptotic pathway.

### 2.3. Anticancer Effects of Rh1 Are Associated with the Akt Signaling Pathway and ROS Modulation

**Figure 3:**
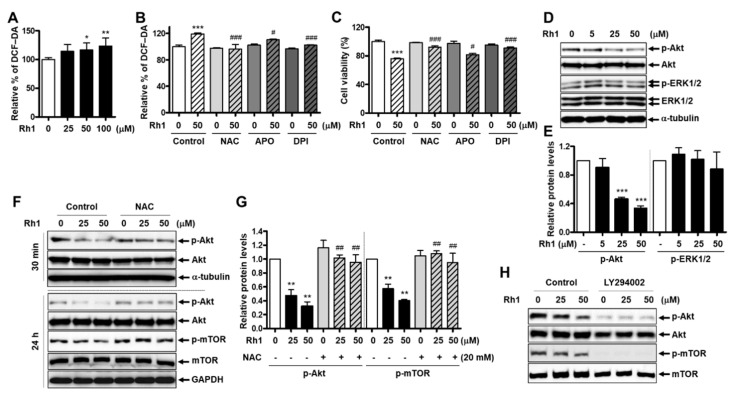
The anticancer effect of Rh1 is associated with the PI3K/Akt pathway and ROS production. (**A**) MCF-7 cells were treated with Rh1 (25 to 100 µM) for 24 h. ROS production was determined using the DCF-DA assay. (**B**,**C**) MCF-7 cells were pretreated with NAC (20 mM), APO (500 µM), or DPI (250 nM) for 1 h, followed by the treatment with Rh1 (50 µM) for 24 h. ROS production was determined using the DCF-DA assay (**B**). Cell viability was measured by the SRB assay (**C**). (**D**,**E**) MCF-7 cells were treated with Rh1 at various concentrations for 30 min. The whole-cell lysates were then used to analyze the kinase activity of ERK1/2 and Akt by Western blotting (**D**). The relative quantification of the protein levels was analyzed using Image J software (**E**). (**F**,**G**) MCF-7 cells were pretreated with NAC (20 mM) for 3 h, followed by the treatment with Rh1 (25 and 50 µM) for 30 min or 24 h. Total cell lysates were subjected to Western blotting. The relative quantification of the protein levels in the experiment with a 24-h treatment of Rh1 was analyzed using Image J software (**G**). (**H**) The cells were pretreated with 5-µM LY294002 for 3 h, followed by the treatment with Rh1 for 24 h. Total cell lysates were subjected to Western blotting. (**A**–**C**,**E**,**G**) Data are expressed as the mean ± SD (*n* = 5), * p < 0.05, ** p < 0.01, and *** p < 0.001 as compared with the control and *# p* < 0.05, ## *p* < 0.01, and ### *p* < 0.001 as compared with the Rh1-treated samples. NAC: N-acetylcysteine, LY294002: PI3K inhibitor, APO: apocynin, and DPI: diphenyleneiodonium.

ROS play an important role in inducing cytotoxicity in cancer cells [4]. Herein, ROS levels in Rh1-treated MCF-7 cells were examined, and the data showed that Rh1 triggered ROS generation in MCF-7 cells in a dose-dependent manner (Figure 3A). There are a variety of sources of ROS, such as NADPH oxidases (NOX), mitochondrial electron transport chain, nitric oxide synthases, cytochrome P450 reductase, and xanthine oxidase. Among them, NOX are the main source to generate ROS, and NOX isoforms have been found to be involved in apoptosis, autophagy, and cell cycle regulation in breast cancer cells [24,25]. To investigate whether Rh1-produced ROS are involved in NOX activity, MCF-7 cells were pretreated with the ROS scavenger, N-acetylcysteine (NAC), the nonspecific NOX inhibitor, diphenyleneiodonium (DPI), or the specific NOX2 inhibitor, apocynin (APO). After treatment with 50-µM Rh1 for 24 h, intracellular ROS production was measured using the DCF-CA assay. The results showed that Rh1-increased ROS production was rescued by NAC, DPI, and APO. The percentage of ROS decreased by DPI or NAC was similar, but APO partially reversed the effect of Rh1 (Figure 3B). The MCF-7 cell viability was further confirmed using these inhibitors, and Rh1-induced cell death was significantly restored by NAC, DPI, and APO at a ratio consistent with the ROS production data in Figure 3B (Figure 3C). This suggests that NOX activities play a key role in Rh1-induced ROS production.

Additionally, the PI3K and MAPK pathways play critical roles in promoting the proliferation of cancer cells [16]. To clarify the mechanisms responsible for the inhibitory effect of Rh1 on MCF-7 and HCC1428 cell viability, PI3 kinase and MAP kinase activity were analyzed after exposure to Rh1 for 30 min. Compared to the control sample, Rh1-treated cells remarkably decreased the phosphorylation of Akt but not ERK1/2 (Figure 3D,E, and Figure S2). Involvement of the PI3K/Akt pathway in the antitumor effect of Rh1 in MCF-7 cells was confirmed using a PI3K inhibitor, LY294002 (5 µM), and we found that LY294002 further decreased the phosphorylation of Akt and mTOR compared to the treatment with Rh1 (Figure 3H). 

Next, we investigated whether ROS would regulate the PI3K pathways. NAC pre-incubation remarkably rescued the suppression of Akt and mTOR phosphorylation induced by Rh1 (Figure 3F,G). These data indicate that the anticancer effect of Rh1 is involved in the PI3K pathway and is regulated by ROS. 

### 2.4. Cell Cycle Arrest and Apoptosis Induced by Rh1 Is Associated with the ROS-Mediated Akt Pathway

**Figure 4:**
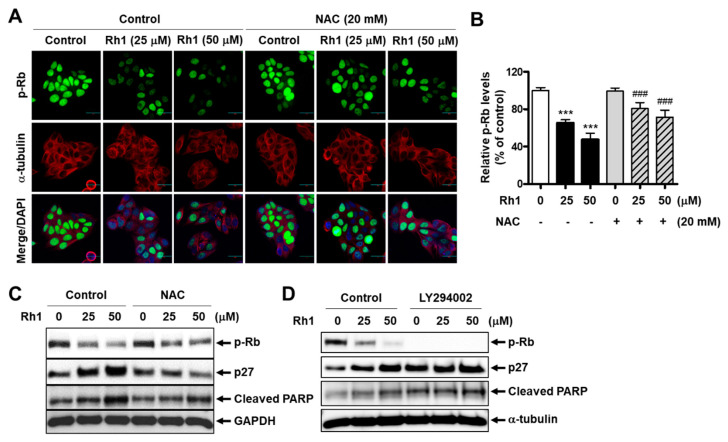
Cell cycle arrest and apoptosis induced by Rh1 is associated with the ROS-mediated Akt pathway in MCF-7 cells. (**A**,**B**) The cells were pretreated with NAC (20 mM) for 3 h, followed by the treatment with Rh1 for 24 h. An immunofluorescence analysis was performed after fixing and immunostaining. Images (**A**) are shown at ×200 magnification. Scale bar indicates 30 μm. The relative expression of phosphorylated Rb (**B**) represents the mean ± SD (*n = 6*), *** p < 0.001 compared with the control sample and ### *p* < 0.001 compared with the Rh1-treated sample, respectively. (**C**,**D**) The cells were pretreated with 20-mM NAC (**C**) or 5-µM LY294002 (**D**) for 3 h, followed by the treatment with Rh1 for 24 h. Total cell lysates were subjected to Western blotting. NAC: N-acetylcysteine and LY294002: PI3K inhibitor.

To determine whether Rh1-induced cell apoptosis and cell cycle arrest would mediate the role of ROS, MCF-7 cells were pre-incubated with 20-mM NAC before exposure to Rh1 for 24 h. An immunofluorescence analysis revealed that Rh1 (25 and 50 µM) significantly inhibited the phosphorylation of Rb in MCF-7 cells, whereas a pretreatment with 20-mM NAC recovered this effect (Figure 4A,B). Consistently, the Rh1-induced upregulation of p27 expression and downregulation of Rb phosphorylation were rescued by the presence of NAC (Figure 4C). In addition, ROS inhibition reduced the elevated protein expression of cleaved PARP caused by Rh1 (Figure 4C). Furthermore, the role of the PI3K pathway in Rh1-produced cell apoptosis and cell cycle arrest in MCF-7 cells was confirmed by using LY294002. The cells were pretreated with 5-µM LY294002 for 3 h and then exposed to Rh1 for 24 h. As seen in Figure 4D, inhibition of the PI3K pathway by LY294002 further increased the protein expression of cleaved PARP and p27 and further decreased the phosphorylation of p-Rb compared to the sample treated with Rh1 alone. Taken together, these data indicate that Rh1-induced cell apoptosis and cell cycle arrest are associated with the PI3K pathway and ROS modulation.

### 2.5. Rh1 Stimulates Autophagy in BC Cells

**Figure 5:**
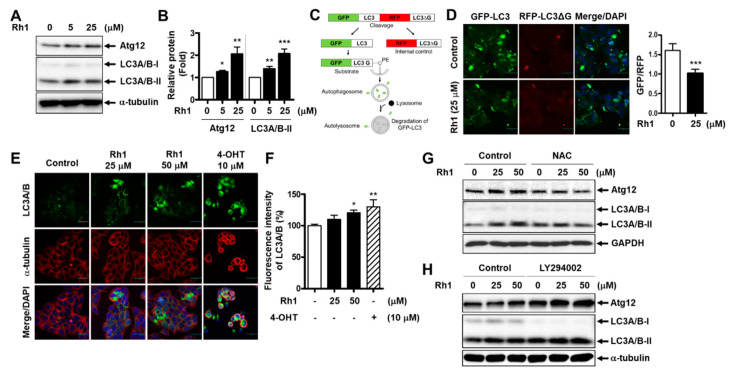
Rh1 induces autophagy in MCF-7 cells. (**A**,**B**) The cells were treated with Rh1 for 24 h. Total cell lysates were then analyzed using Western blotting (**A**). The relative quantification of protein levels was analyzed using Image J software (**B**). (**C**) The scheme presents the principle of the dual GFP/RFP-LC3 autophagy flux sensor system. (**D**) MCF-7 cells were transduced with pMRX-IP-GFP-LC3-RFP-LC3ΔG retroviral vectors for 30 h and then treated with Rh1 (25 µM) for 14 h. The cells were then observed under a laser scanning confocal spectral microscope. Images are shown at ×200 magnification. Scale bar indicates 30 μm. The relative GFP/RFP ratio is represented as the mean ± SD (*n* = 5). *** p < 0.001 as compared with the control. (**E**,**F**) The cells were treated with Rh1 (25 and 50 µM) or 4-OHT (10 µM) for 24 h. An immunofluorescence analysis was performed after fixing and immunostaining. Images (**E**) are shown at ×200 magnification. Scale bar indicates 30 μm. The relative fluorescence intensity of LC3A/B (**F**) are represented as the mean ± SD (*n* = 5), * p < 0.05 and ** p < 0.01 as compared with the control. (**G**,**H**) The cells were pretreated with 20-mM NAC (**G**) or 5-µM LY294002 (**H**) for 3 h, followed by the treatment with Rh1 for 24 h. Total cell lysates were subjected to Western blotting. 4-OHT: 4-hydroxytamoxifen, NAC: N-acetylcysteine, LY294002: PI3K inhibitor, GFP: green fluorescent protein, and RFP: red fluorescent protein.

To determine whether the anticancer effect of Rh1 could be related to autophagy induction, MCF-7 cells were pretreated with 10-µM 3-methyladenine (3-MA), an autophagy inhibitor, before treatment with Rh1 for 24 h. Cell viability was then analyzed through the SRB assay. The data showed that Rh1-decreased cell viability was partially reversed by 3-MA, indicating that Rh1-induced cell death is related to autophagy (Figure S3). Next, the expression of autophagy-related proteins was examined in Rh1-treated cells. The Western blot analysis showed that Rh1 treatment increased the expression of Atg12 and LC3A/B-II compared to that in the control groups in MCF-7 cells (Figure 5A,B). Notably, to demonstrate the effects of the compound on the autophagic flux, a dual green fluorescent protein (GFP)/red fluorescent protein (RFP)-LC3 autophagy flux sensor system was used. This system is based on the expression of the GFP-LC3-RFP-LC3ΔG fusion protein, which consists of GFP fused to wild-type LC3 and RFP fused to the LC3 Gly120 mutant [26]. This fusion protein is intracellularly cleaved by ATG4 proteases into equimolar ratios. GFP-LC3 is localized in autophagosomes and is partly degraded in autolysosomes due to the induction of autophagy. Meanwhile, the mutated RFP-LC3ΔG cannot be conjugated to autophagosomes, which is not affected by autophagy, thus serving as an internal control (Figure 5C) [26]. There was an overall decrease in the relative ratio of GFP/RFP in GFP-LC3-RFP-LC3ΔG reporter-expressing MCF-7 cells treated with Rh1, as compared to the untreated cells (Figure 5D). This indicates an enhancement of autophagy flux in cells exposed to Rh1. In addition, the increased expression of LC3A/B in MCF-7 cells treated with Rh1 was confirmed by an immunofluorescence analysis (Figure 5E,F). 

Since we found that the inhibition of Akt phosphorylation induced by Rh1 involved ROS production, the effect of Rh1 on the expression of autophagy-related proteins was further examined in the presence of NAC. Figure 5G shows that Atg12 and LC3A/B-II expression elevated by Rh1 were rescued by NAC, suggesting that autophagy induction by Rh1 is also associated with ROS production. Additionally, the role of the PI3K/Akt pathway in autophagy induction in Rh1-treated MCF-7 cells was elucidated. The cells were pretreated with 5-µM LY294002 before treatment with Rh1 and subjected to Western blotting. Figure 5H shows that inhibition of the PI3K pathway by LY294002 enhanced the effect of Rh1 on the expression of Atg12 and LC3A/B-II. Altogether, these findings documented the ability to induce the autophagy of Rh1 in MCF-7 cells, which is correlated with the inhibition of the PI3K/Akt pathway and ROS generation.

### 2.6. Rh1 Showed a Competitive Effect with an Estrogenic Agent on BC Cells

**Figure 6:**
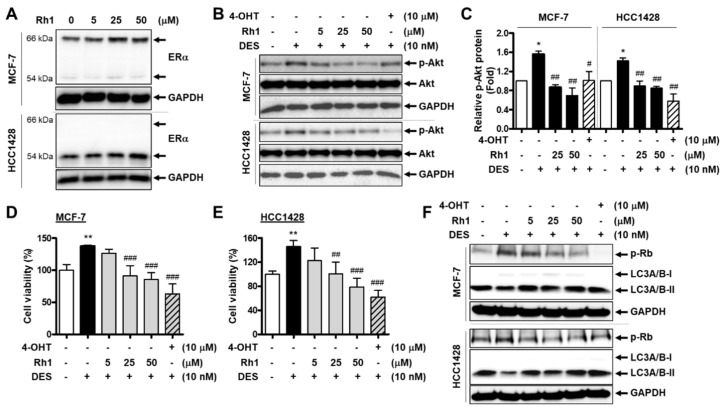
Rh1 showed a competitive effect with an estrogenic agent on BC cells. (**A**) MCF-7 cells and HCC1428 cells were treated with different concentrations of Rh1 (5, 25, and 50 µM) for 24 h. Total cell lysates then analyzed the estrogen receptor alpha (Erα) protein expression using Western blotting. (**B**,**C**) MCF-7 cells and HCC1428 cells were treated with 10-nM DES in the presence or absence of Rh1 (5, 25, and 50 µM) or 10-µM 4-OHT for 30 min. Total cell lysates were then analyzed using Western blotting (**B**). The relative quantification of protein levels (**C**) was analyzed using Image J software. (**D**,**E**) MCF-7 cells (**D**) and HCC1428 cells (**E**) were treated with 10-nM DES in the presence or absence of Rh1 (5, 25, and 50 µM) or 10-µM 4-OHT for 24 h. The 3-(4, 5-dimethylthiazole-2-yl)-2, 5-diphenyltetrazolium bromide (MTT) assay was used to analyze the cell viability. (**F**) MCF-7 cells and HCC1428 cells were treated with 10-nM DES in the presence or absence of Rh1 (5, 25, and 50 µM) or 10-µM 4-OHT for 12 h. Total cell lysates were then analyzed using Western blotting. (**C**–**E**) Data are presented as the mean ± SD (*n = 3*), * p < 0.05 and ** p < 0.01 compared with the control sample and # *p* < 0.05, ## *p* < 0.01 and ### *p* < 0.001 compared with the DES-treated sample, respectively. 4-OHT: 4-hydroxytamoxifen and DES: diethylstilbestrol.

Rh1 has been reported to exhibit weak estrogenic activity in BC cells [27]. Consistently, we found that Rh1 increased estrogen receptor alpha (ERα) protein expression in both MCF-7 and HCC1428 cells (Figure 6A). Therefore, we investigated whether this ginsenoside could exert a competitive effect in the presence of an estrogenic agent. Since the binding between estrogen and plasma membrane-associated ERα rapidly activates the PI3K/Akt pathway in BC cells [28], we first examined the effect of Rh1 on Akt activation induced by diethylstilbestrol (DES), a synthetic form of estrogen, in MCF-7 and HCC1428 cells. The cells were treated with 10-nM DES in the presence or absence of various concentrations of Rh1 for 30 min. 4-OHT was used as a positive control for the competitive effect on ERα activation. The increase in Akt phosphorylation induced by DES was completely inhibited by the presence of Rh1, and it further decreased compared to that in the control (Figure 6B,C). Notably, after 24 h of exposure, DES significantly augmented the viability of MCF-7 cells and HCC1428 cells, whereas Rh1 dramatically suppressed these effects (Figure 6D,E). Consistent with the effect of Rh1 on the cell cycle and autophagy regulation in previous experiments, the presence of Rh1 completely reversed the increased Rb phosphorylation and the decreased LC3A/B-II protein expression caused by DES (Figure 6F). Taken together, these data suggest that Rh1 exerts a competitive effect to inhibit BC cell proliferation in response to estrogenic agents. 

### 2.7. Rh1 Exerted Antitumor Activities in a Xenograft Model

**Figure 7:**
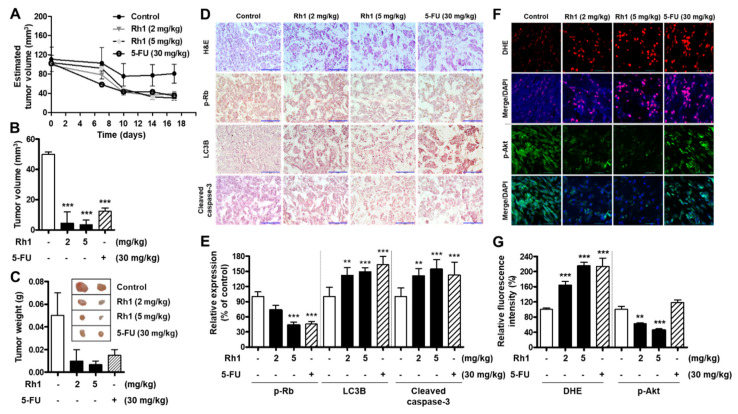
Rh1 exerted antitumor activities in a xenograft model. (**A**) Estimated tumor volumes were measured every three days after the implantation using caliper measurements and calculated with the following formula: volume = 0.5 × (length × width^2^). (**B**,**C**) Sixteen days after the administration of Rh1 (2 and 5 mg/kg), 5-FU (30 mg/kg), or DMSO, the xenograft tumors were collected; the average ± SD tumor volume (**B**) and tumor weight (**C**) were analyzed. *** p < 0.001 compared with the control sample. The images showed the tumors generated by different groups (**C**). (**D**) Hematoxylin and eosin staining (upper panel) and an immunohistochemistry analysis of p-Rb, LC3B, and cleaved caspase-3. Scale bar indicates 100 μm. (**E**) Quantified bar graph indicates p-Rb, LC3B, and cleaved caspase-3 expression in tumor tissues. (**F**) Dihydroethidium (DHE) staining (two upper panels) and immunofluorescence staining of Akt phosphorylation (two lower panels) in tumor tissues. (**G**) Quantified bar graph indicates the relative fluorescence levels of DHE and Akt phosphorylation expression in tumor tissues. (**A**–**C**,**E**,**G**) Data are expressed as the mean ± SD (*n* = 5), ** p < 0.01 and *** p < 0.001 as compared with the control. 5-FU: 5-fluorouracil and DMSO: dimethyl sulfoxide.

To further confirm the anticancer effect of Rh1 in vivo, xenograft tumors were established by transplanting MCF-7 cells into the flanks of nude mice. The xenograft mice were treated with Rh1 (2 and 5 mg/kg) or 5-fluorouracil (5-FU; 30 mg/kg) every three days for 16 days. As a result, the drug treatment led to a significant decrease in tumor size (Figure 7A–C) and attenuated cell growth, as indicated by the decrease in cell population, which was recorded via hematoxylin and eosin (H&E) staining of the tumor sections (Figure 7D, upper panel). Additionally, as seen through immunohistochemical staining, there was a decrease in the levels of phosphorylated Rb protein expression but increased levels of LC3B and cleaved caspase-3 in the tumors of Rh1-treated and 5-FU-treated mice, as compared with the vehicle-treated group (Figure 7D,E). Consistently, the dihydroethidium (DHE) assay showed a remarkable increase in ROS production in the tumors of treated mice (Figure 7F, two upper panels, and Figure 7G). Furthermore, Akt phosphorylation in the tumors of Rh1-treated mice was dose-dependently decreased, as shown by immunofluorescence staining (Figure 7F, two lower panels, and Figure 7G). These results indicate that Rh1 possesses potential antitumor properties in BC in vivo and further confirmed the in vitro results, in which Rh1 induced autophagy, apoptosis, and cell cycle arrest through ROS-mediated Akt inhibition.

## 3. Discussion

Natural bioactive compounds are potential sources to develop drugs for the treatment of various diseases. In particular, a number of ginsenosides have been documented to exhibit various pharmacological effects, including anticancer activities [5,6,29,30]. Among them, Rh1 has been reported to have an ability to suppress several cancer cell lines, such as leukemia P388 cells, lung cancer A549 cells, and cervical cancer HeLa cells [19]. The effective dose of Rh1 differed among the cell lines. For instance, the IC_50_ value of Rh1 in P388 cells was 37 µM [31]. Meanwhile, Rh1 inhibited approximately 30% of the cell viability of A549 cells at 100 µM [23] and suppressed approximately 25% of the cell viability of HeLa cells at 40 µM [32]. However, whether this compound can inhibit BC has not yet been reported. We found that Rh1 exerted anticancer effects on MCF-7 and HCC1428 cells through various mechanisms, including the induction of apoptosis and autophagy and the regulation of cell cycle progression.

The inhibition of cell survival and proliferation through apoptotic pathways is a promising therapeutic strategy for cancer therapy [9]. In the current study, we found that Rh1 increased apoptosis, as demonstrated by PI/Hoechst double staining, Western blotting, and an immunofluorescence analysis with confocal microscopy. In fact, through Hoechst staining, Rh1 was found to dramatically increase the number of apoptotic cells with condensed and fragmented nuclei (Figure 1B). In addition, the protein levels of cleaved PARP and cleaved caspase-3 were significantly increased by the treatment with Rh1 (Figure 2A–C). Besides the induction of apoptosis, the modulation of cell cycle progression is another mechanism that controls cell proliferation in cancer therapy. In our study, Rh1 induced p27 protein expression, whereas it reduced proteins related to the G1 phase, such as Rb phosphorylation, cyclin D1, cyclin D3, CDK2, CDK4, and CDK6, to prevent the shift of the cell cycle from the G1 to S phase (Figure 2D–G). 

In previous studies, Rh1 has been reported to exert an inhibitory effect on the migration and invasion of several cancer cells, such as monocytic leukemia cells, human hepatocellular carcinoma cells, and colorectal cancer cells, mainly through the inactivation of the MAPK pathway [21,30]. Interestingly, in our study, the PI3K/Akt pathway, but not the MAPK pathway, was found to be the key pathway associated with the antitumor activity of Rh1 in BC cells. The PI3K signaling pathway is commonly activated in cancer cells and is a potent target for cancer therapy [18]. Furthermore, the PI3K/Akt/mTOR pathway has been known to be pivotal in autophagy regulation [14]. In our study, the treatment with Rh1 increased the expression of Atg12 and LC3A/B-II in a dose-dependent manner, as well as enhanced the autophagy flux in MCF-7 cells (Figure 5). Additionally, Rh1 significantly decreased the phosphorylation of Akt and mTOR in MCF-7 cells (Figure 3D,H). Notably, the inhibition of Akt kinase activity using LY294002 further enhanced the expression of Atg12 and LC3A/B-II protein expression compared to the corresponding control (Figure 5H). Therefore, in agreement with the previous studies, our study showed that Rh1-induced autophagy is regulated by the PI3K/Akt/mTOR pathway. In addition to autophagy, in our study, the effect of Rh1 on cell cycle arrest and apoptosis was also demonstrated to be involved in the PI3K/Akt pathway. Indeed, the inhibition of this pathway using LY294002 further enhanced the effect of Rh1 on molecules related to cell cycle arrest (p-Rb and p27) and apoptosis (cleaved PARP) (Figure 4D). This phenomenon is also consistent with various findings, in which apoptosis, cell cycle arrest, and autophagy share common regulatory pathways, including the PI3K/Akt signaling pathway [33,34,35]. Additionally, it has been reported that cell cycle arrest mediates apoptosis in cancer cells by various anticancer agents [36], and autophagy has the ability to increase apoptosis [37]. Therefore, the PI3K/Akt pathway may be one of the pathways that links the three pathways in Rh1-treated MCF-7 cells (apoptosis, cell cycle arrest, and autophagy). 

Furthermore, it has been well-established that ROS mediates apoptosis, autophagy, and cell cycle arrest in various human cancer cell lines [4]. In this study, we found that Rh1 increased ROS production in MCF-7 cells. Interestingly, NAC remarkably reversed Rh1-induced cell death, restored the decreased Akt phosphorylation, and phosphorylated Rb in Rh1-treated MCF-7 cells (Figure 3C,F and Figure 4C). Additionally, this ROS scavenger reduced the elevated levels of cleaved PARP, Atg12, LC3A/B-II, and p27 induced by Rh1 in MCF-7 cells (Figure 4C and Figure 5G). These results suggest that Rh1-induced apoptosis, autophagy, and cell cycle arrest in MCF-7 cells are closely associated with ROS generation, which may be an upstream factor that modulates the PI3K/Akt pathway. Furthermore, DPI, a NOX inhibitor, recovered Rh1-induced ROS production and Rh1-decreased cell viability in MCF-7 cells in the same proportion as NAC, indicating that Rh1-produced ROS accumulation in MCF-7 cells is associated with NOX activity (Figure 3B,C). Besides, DPI is a nonspecific NOX inhibitor, while APO mainly acts as a NOX2 inhibitor [38,39]. Therefore, the recovery effects of APO on the effect of Rh1 was lower than that of DPI (Figure 3B,C). In addition, to inhibit NOX activity in MCF-7 cells, previous studies used DPI at concentrations of 10 µM or 5 µM [25,40]. However, since we found that those concentrations of DPI are relatively toxic for the cells after 25 h of treatment in our system, the MTT assay was performed to investigate the doses of DPI. Subsequently, 250 nM of DPI was used to inhibit NOX activity in our study, because this concentration did not show any significant change in the viability of MCF-7 cells (Figure S4). Furthermore, it has been reported that ROS accumulation promotes PTEN and activates AMPK, which is associated with the inhibition of the PI3K/Akt pathway [41,42]. Additionally, the activation of AMPK and PTEN is related to apoptosis and autophagy induction [41,43]. In our study, ROS-mediated PI3K/Akt inhibition promoted apoptosis and autophagy. Altogether, PTEN and AMPK activation may be the links between ROS and PI3K inhibition in Rh1-treated MCF-7 cells. 

Consistent with the in vitro data, the 5-mg/kg Rh1-treated group showed increased ROS generation in tumor tissues, similar to the 30-mg/kg 5-FU-treated group. Additionally, Rh1 inhibited Akt phosphorylation in tumor tissues in a dose-dependent manner (Figure 7F). However, 5-FU slightly increased the phosphorylation of Akt in tumors, and the anticancer mechanisms of 5-FU in MCF-7 cells may not be related to the inhibition of the PI3K/Akt pathway. Several reports have revealed that Akt is activated in 5-FU-treated MCF-7 cells [44,45]. 

ERα plays a key role in regulating the cell growth of ER-positive BC cells [28]. In addition to the promotion of growth and survival gene expression, the binding of estrogen to ERα also leads to the activation of the PI3K/Akt signaling pathway [28]. In our study, Rh1 was shown to reverse the stimulation of cell growth induced by a synthetic estrogen, DES, in both MCF-7 and HCC1428 cells (Figure 6D,E). Concomitantly, Rh1 exerted a recovery effect on DES-induced Akt activation (Figure 6B). Consistently, the effect of DES on proteins related to the cell cycle (p-Rb) and autophagy (LC3A/B) was also reversed by the presence of Rh1 (Figure 6F). Altogether, in the presence of an estrogenic agent, Rh1 seems to competitively bind to ERα, leading to the attenuation of estrogen stimulation. These results may be attributed to the weak estrogenic property, as revealed in a previous study [27] and confirmed in Figure 6A. Interestingly, Rh1 not only rescued the stimulation of DES on cell growth and Akt activation but, also, further decreased the cell viability and Akt phosphorylation as compared with the control. This may result from the inhibitory effect of Rh1 via ROS-mediated Akt signaling, which may be independent from the competitive effect on ERα binding. 

Among the three ER-positive BC cell lines (MCF-7, HCC1428, and BT474), the BT474 cell line was insensitive to Rh1 (Figure 1A). This phenomenon may be attributed to the differences in characteristics of the BC cell subtypes. MCF-7 and HCC1428 cells are HER2-negative BC, while BT474 cells are HER2-positive BC [46]. HER2 overexpression has been found to be associated with enhanced cell proliferation and resistance to therapies [47]. In this manner, Rh1 may be more effective in HER2-negative BC cells rather than in HER2-postive BC cells. In addition, it has been reported that patients’ expressed wild-type p53 tumors are more sensitive to chemotherapy than those with mutant p53 tumors [48]. HCC1428 cells are known as mutations in the tumor suppressor p53. Therefore, in our study, HCC1428 cells were less sensitive to Rh1 than MCF-7 cells, possibly because of the difference in p53 status between MCF-7 and HCC1428 cells. Additionally, this suggests that Rh1 may also target the tumor suppressor p53 in MCF-7 cells, which is worth investigating in future studies.

Notably, it has been reported that inflammation can promote cancer progression and facilitate tumorigenesis in all stages [49]. We previously reported that the Rh1 and Rg2 combination has anti-inflammatory effects and protective effects against liver injury and kidney damage induced by lipopolysaccharides [20,50]. Another study showed that Rh1 plays a key role in immune regulation [19]. Therefore, the anti-inflammatory effect and the role in the immune regulation of Rh1 possibly support and enhance the anticancer effect of Rh1 when applied to cancer patients. In addition to its low toxicity in normal cells and animal models [29,50,51,52], Rh1 can serve as a good candidate for BC therapy.

## 4. Materials and Methods 

### 4.1. Materials

Rabbit anti-phospho-Akt ser473 (#9271), rabbit anti-Akt (#9272), rabbit anti-phospho-ERK1/2(#4370), rabbit anti-ERK1/2 (#4695), rabbit anti-phospho-mTOR (#5536T), rabbit anti-mTOR (#2983T), rabbit anti-PARP (#9532), rabbit anti-cleaved caspase-3 (Asp175) (# 9664), anti-phospho-Rb (Ser807/811) (#8516), rabbit anti-Atg12 (#2010), rabbit anti-LC3A/B (#4108), rabbit anti-LC3B (#3868), rabbit anti-p27 kip 1 (#3686), rabbit anti-Cyclin D1 (#2978), mouse anti-Cyclin D3 (#2936), and rabbit anti-CDK2 (#2546) antibodies were purchased from Cell Signaling Technology, Inc. (Danvers, MA, USA). Mouse anti-p53 (#47698) and Z-VAD-FMK (#3067) were purchased from Santa Cruz Biotechnology Inc. (Dallas, TX, USA). Rabbit anti-GAPDH (#LF-PA0018) was purchased from AbFrontier (Seoul, Korea). EndoFectin™ Max Transfection Reagent (EFM1004) was purchased from GeneCopoeia (Rockville, MD, USA). Mouse anti-α-tubulin antibody, anti-mouse IgG (#T5393), crystal violet (#C0775), 2′,7′-dichlorodihydrofluorescein diacetate (DCF-DA), sulforhodamine B sodium salt (SRB, #S1402), trichloroacetic acid (TCA, #T6399), H&E, and Rh1 were purchased from Sigma-Aldrich (St. Louis, MO, USA). Mounting medium with DAPI was purchased from Vector Laboratories, Inc. (#Z0215, Burlingame, CA, USA). Phosphate-buffered saline (PBS; #EBA-1105) was purchased from Elpisbio (Daejeon, South Korea). 3-(4, 5-dimethylthiazole-2-yl)-2, 5-diphenyltetrazolium bromide (MTT; #M6494), propidium iodide (PI; #P1304MP), and Hoechst 33342 (#H3570) were purchased from Invitrogen (Carlsbad, CA, USA). Muse cell cycle reagent (#MCH100106) was purchased from Millipore (Billerica, MA, USA). Bovine serum albumin (#160069) was obtained from ICN Biomedicals, Inc. (Costa Mesa, CA, USA). Dulbecco’s modified Eagle’s medium (DMEM; #11963–092), Roswell Park Memorial Institute Medium (RPMI; 11875093)-1640, and fetal bovine serum (FBS; #10082147) were purchased from Gibco (Carlsbad, CA, USA). Diphenyleneiodonium chloride (DPI; #0504), apocynin (APO; #4663), and 3-methyladenine (3-MA; #3977) were purchased from Tocris Bioscience (Ellisville, MO, USA). pMRX-IP-GFP-LC3-RFP-LC3ΔG plasmid (Addgene plasmid #84572) was a gift from Noboru Mizushima.

### 4.2. Cell Culture 

Human mammary carcinoma cell lines MCF-7 (AHTB-22^TM^) and HCC1428 (CRL-2327™) were purchased from the American Type Culture Collection (ATCC, Manassas, VA, USA). BT474 cells were purchased from the Korean Cell Line Bank (Seoul, Korea). MCF-7 cells were maintained in DMEM containing 10% FBS and 100-U/mL penicillin and streptomycin. HCC1428 cells were cultured in RPMI-1640 supplemented with 10% FBS and 100-U/mL penicillin and streptomycin. BT474 cells were cultured in RPMI-1640 with L-glutamine (300 mg/L); 25-mM HEPES; and 25-mM sodium bicarbonate supplemented 10% FBS and 100-U/mL penicillin and streptomycin. The cells were incubated at 37 °C in a humidified atmosphere of 5% CO_2_ (HERAcell 150i, hermo Electron Corp., Waltham, MA, USA). 

### 4.3. Sulforhodamine B (SRB) Assay

Cell proliferation was assessed by SRB assay according to the previous report [53]. Briefly, cells were seeded at a concentration of 1.9 × 10^4^ cells/well in 96-well plates and incubated with various concentrations of Rh1 for 72 h. After the treatment, cold 10% (wt/vol) TCA was added to each well to reach the final TCA concentration of 3.3% (wt/vol) and incubated at 4 °C for 1 h. The plates were then washed with slow-running tap water and air-dried at room temperature (RT) before being stained with 0.057% SRB for 30 min at RT. The plates were quickly rinsed four times with 1% acetic acid and air-dried at RT. The dye was solubilized with a 10-mM Tris base solution (pH 10.5). Finally, absorbance was measured at 510 nm on a microplate reader (Tecan, Männedorf, Switzerland). 

### 4.4. MTT Assay

The cells were seeded in 96-well at a density of 1 × 10^4^ cells per well. After 24 h of incubation, the cells were performed with the indicated treatment. Cell viability was then determined according to our previous report [50].

### 4.5. Western Blot

After the treatment, MCF-7 cells were washed with PBS, and total cell lysates were prepared using 2× sodium dodecyl sulfate (SDS) lysis buffer, including 1-M Tris-HCl (pH 7.4), 25% glycerol, 10% SDS, 5% 2-mercaptoethnol, and 1% bromophenol blue. The protein extracts were then resolved by SDS-PAGE and the indicated proteins analyzed as previously described [54]. Original Western Blot figures can be found from file S1.

### 4.6. Propidium Iodide (PI)/Hoechst 33342 Double Staining

MCF-7 cells were treated with different concentrations of Rh1 for 24 h. After the treatment, the cells were washed with PBS before stained with PI (10 µM) at 37 °C for 10 min. The cells were then washed with PBS, stained with Hoechst 33342 (5 µg/mL) for 10 min, and fixed in 4% formaldehyde for 10 min. After being washed with PBS, the cells were mounted with Prolong Gold Antifade Reagent (#P36934, Molecular Probes, Inc., Eugene, OR, USA). Subsequently, the images were taken, and a number of cells were quantified using a laser scanning confocal spectral microscope (K1-Fluo, Nanoscope systems, Daejeon, Korea), and a total of 6 fields per sample were counted. Hoechst 33342 (blue fluorescence) stained the nuclei of both live cells and dead cells, whereas PI (red fluorescence) only stained the nuclei of dead cells. Therefore, the cells stained with PI were indicated as dead cells.

### 4.7. Generation of pMRX-IP-GFP-LC3-RFP-LC3ΔG-Expressing Cells and Measurement of the Autophagic Flux

Platinum-E cells were transfected with pMRX-IP-GFP-LC3-RFP-LC3ΔG plasmid by using Endofectin reagent according to the manufacturer’s instructions. Retroviral supernatant was harvested after 48 h of transfection. 

For determination of the autophagic flux, MCF-7 cells were seeded on a cover glass bottom plate (#30206, SPL, Pocheon-so, South Korea) overnight at 37 °C and 5% CO_2_. The cells were transduced with pMRX-IP-GFP-LC3-RFP-LC3ΔG retroviral vectors for 30 h and then treated with Rh1 (25 µM) for 14 h. Subsequently, the cells were fixed with 4% formaldehyde for 10 min and mounted with DAPI mounting solution. The cells were then observed under a laser scanning confocal spectral microscope (K1-Fluo, Nanoscope systems, Daejeon, South Korea).

### 4.8. Immunofluorescence Assay

Immunofluorescence staining was performed as previously described [50]. Briefly, MCF-7 cells were cultured on a cover glass bottom plate overnight at 37 °C and 5% CO_2_. After the treatment, the cells were fixed with 4% formaldehyde for 10 min. The cells were permeated with 0.2% Triton X-100 for 10 min at RT before being blocked with 3% bovine serum albumin for 30 min at RT. Next, the cells were incubated with primary antibody, as indicated overnight at 4 °C, and then incubated with fluorescent secondary antibody for 1 h at RT. Subsequently, the fluorescent images were taken by a laser scanning confocal spectral microscope.

### 4.9. Clonogenic Assay

A clonogenic assay was performed according to the previous protocols [55]. Briefly, MCF-7 cells were seeded into 6-well plates at a density of 500 cells per well. After 2 h for attachment, cells were treated with various concentrations of Rh1 for 10 days. At the end of the treatment, the formed cell colonies were carefully washed with PBS and incubated with 2 mL of 4% formaldehyde and 0.5% crystal violet mixture for 30 min. Finally, the plates were washed and air-dried at RT. The colony, which is defined to constitute at least 50 cells, was manually counted.

### 4.10. Measurement of Intracellular ROS Levels

After the treatment, MCF-7 cells were loaded with 10-µM DCF-DA in PBS for 30 min at 37 °C. Afterwards, cells were washed and maintained in PBS. Fluorescence was monitored using a microplate fluorometer (Tecan, Männedorf, Switzerland) using wavelengths of 485 and 530 nm for excitation and emission, respectively.

### 4.11. Flow Cytometry Analysis of Cell Cycle 

At the end of the treatment, samples collected were centrifuged at 2000 rpm for 1 min at 4 °C and washed with PBS. The obtained pellets were fixed in chilled 70% ethanol and then kept at −20 °C for 6 h. After being fixed, cells were washed with PBS, and the cell pellets were resuspended in 100 μL of Muse cell cycle reagent and incubated for 30 min at RT in the dark. The cell cycle distribution was then assayed using a Muse Cell Analyzer (Merck, Millipore, Billerica, MA, USA). Flow cytometry data were gated to exclude doublets and debris. The exclusion of doublet and debris was assessed by adjusting the height or width against the forward-scatter or side-scatter areas. Doublets double the area and width values of single cells while the height is approximately the same. Meanwhile, small debris has low scatter, whereas large debris area is greater than the range of one or both parameters.

### 4.12. In Vivo MCF-7 Cell Xenograft Nude Mice Model

Xenograft models in nude mice were established on female BALB/c nu/nu mice, which were obtained from Raonbio Co., Ltd. (Yongin-si, Gyeonggi-do, Korea). The mice were acclimated for one week before the experiments and were fed with standard mice feed under 23 ± 2 °C and 12:12-h dark-to-light conditions. All animal studies were performed with approval from the Institutional Animal Care and Use Committee of Chungnam National University, Daejeon, Korea (201912A-CNU-202).

To establish a xenograft model, MCF-7 cells (1.0 × 10^7^ cells) in serum-free DMEM were mixed with an equal ratio of Matrigel (#354248, Corning Inc., Corning, NY, USA) and injected subcutaneously into the right flanks of BALB/c nude mice. The tumor volume and body weight were recorded every three days after tumor injection. The mice were randomly divided into 4 groups (*n* = 5). The mice in the control group were intraperitoneally injected with 5% dimethyl sulfoxide in PBS. The mice in the positive group were injected with 5-FU (30 mg/kg). The mice in the Rh1-treated groups were treated with 2 or 5 mg/kg of Rh1. The drugs were intraperitoneally administered every three days for 16 days. At the end of the test, the mice were sacrificed, and the tumors were harvested. 

### 4.13. Histological Analysis

The fixed tumors were embedded in paraffin and were sectioned to a thickness of 5 μm using a rotary microtome. Each sectioned slide was stained with H&E. Digital images were captured using a light microscope (Olympus IX71, Olympus Optical Co. Ltd, Tokyo, Japan).

### 4.14. Detection of Reactive Oxygen Species in Tumor Tissues through Dihydroethidium (DHE) Staining

After deparaffinization and rehydration, the 5-μm-thick sections were stained with 10-µM DHE for 30 min. The slides were then washed with PBS and mounted with DAPI mounting solution. The sections were analyzed with a laser scanning confocal spectral microscope.

### 4.15. Immunohistochemistry Staining

The 5-μm-thick sections of the tissues were used. After deparaffinization and rehydration, the tissue sections were incubated with 1% hydrogen peroxide for 10 min. Then, 5% bovine serum albumin solution was applied to prevent nonspecific staining. Next, the sections were incubated with primary antibody at RT for 2 h. The biotinylated secondary antibody, the avidin-biotin complex, and the 3,3’-diaminobenzidine chromogens were then applied, respectively. Sections were counterstained with hematoxylin. After dehydration, sections were analyzed using a light microscope (Olympus IX71, Olympus Optical Co. Ltd, Tokyo, Japan).

### 4.16. Statistical Analysis

Statistical analysis was carried out using GraphPad Prism 5 (version 5.02; GraphPad Software Inc., San Diego, CA, USA) and one-way analysis of variance (ANOVA), followed by a Bonferroni multiple comparison. Comparisons between two groups were performed by using Student’s unpaired *t*-test. *p* < 0.05 was considered to be significant. All data were expressed as the mean ± SD. 

## 5. Conclusions

In conclusion, our study, for the first time, demonstrated that Rh1 exerted potential anticancer effects on ER-positive BC, MCF-7, and HCC1428 cells by activating cell apoptosis, cell cycle arrest, and autophagy. Mechanistically, Rh1-mediated ROS generation induced inhibition of the cancer cell survival signaling pathways, including PI3K/Akt/mTOR. Importantly, Rh1 possessed a competitive effect in the presence of DES, a synthetic form of estrogen, thereby inhibiting estrogen-stimulated cell proliferation. 

## Data Availability

Data is contained within the article and supplementary material.

## Supplementary Materials

The following are available online at https://www.mdpi.com/article/10.3390/cancers13081892/s1: Figure S1: Rh1 induced cell apoptosis and cell cycle arrest in HCC1428 cells. Figure S2: Effects of Rh1 on the PI3K/Akt and MAPK pathways in HCC1428 cells. Figure S3: Anticancer effect of Rh1 in MCF-7 cells is involved in apoptosis and autophagy. Figure S4: Effect of diphenyleneiodonium (DPI) on MCF-7 cell viability. File S1: Original Western Blot figures.
