# Supplementary material for: Ginsenoside Rh1 Induces MCF-7 Cell Apoptosis and Autophagic Cell Death through ROS-Mediated Akt Signaling
Source: Cancers (Basel). 2021 Apr 15;13(8):1892. doi: 10.3390/cancers13081892 (PMC8071122; doi:10.3390/cancers13081892)
Supplement: Supplementary file 1 [file cancers-13-01892-s001.zip › cancers-1086602-file S1.pdf]

Whole blot supplementary materials

Figure 2

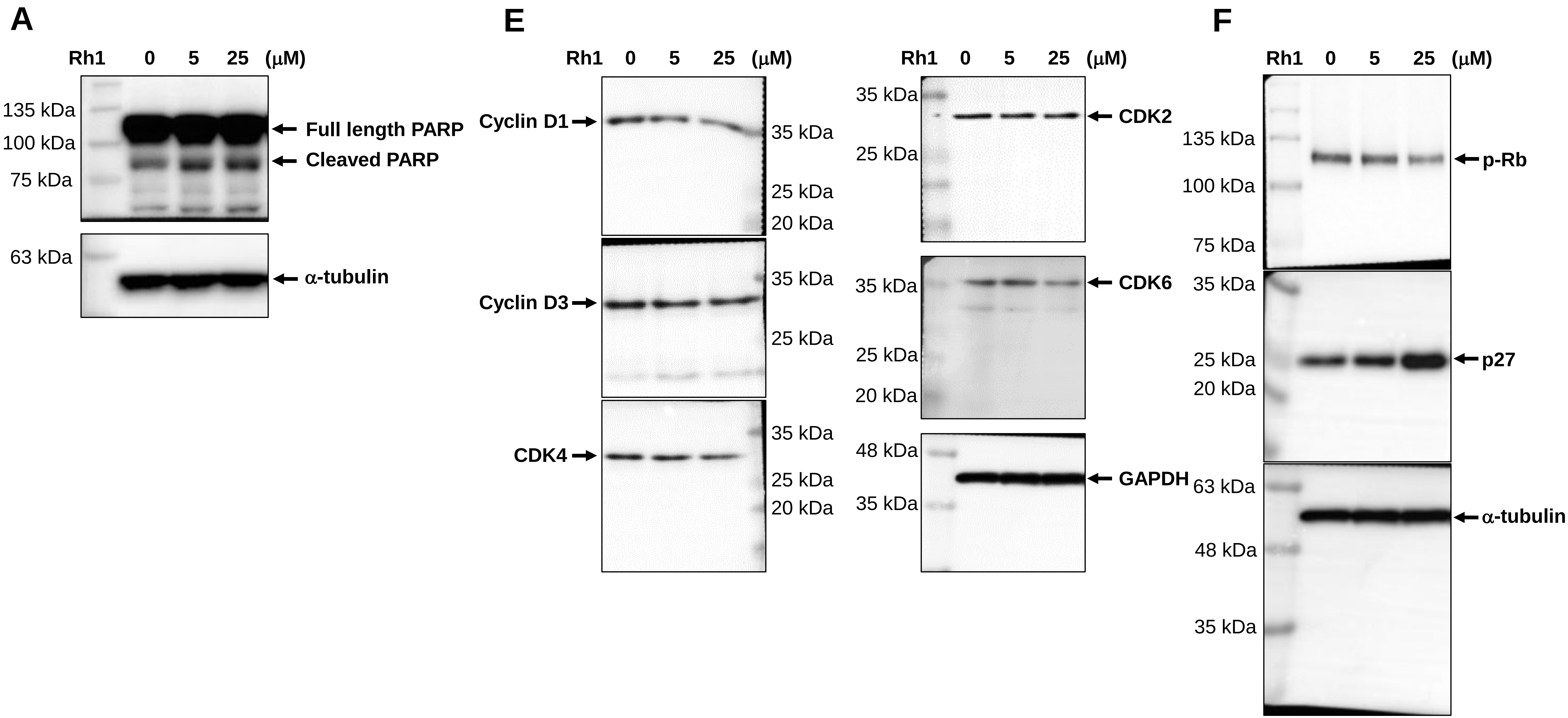

A
Rh1 0 5 25 (μM)
135 kDa
100 kDa
75 kDa
Full length PARP
Cleaved PARP
63 kDa
α-tubulin
E
Rh1 0 5 25 (μM)
Cyclin D1
35 kDa
25 kDa
20 kDa
Cyclin D3
35 kDa
25 kDa
CDK4
35 kDa
25 kDa
20 kDa
Rh1 0 5 25 (μM)
35 kDa
25 kDa
CDK2
35 kDa
25 kDa
20 kDa
CDK6
48 kDa
35 kDa
GAPDH
F
Rh1 0 5 25 (μM)
135 kDa
100 kDa
75 kDa
p-Rb
35 kDa
25 kDa
20 kDa
p27
63 kDa
48 kDa
35 kDa
α-tubulin

Figure 3

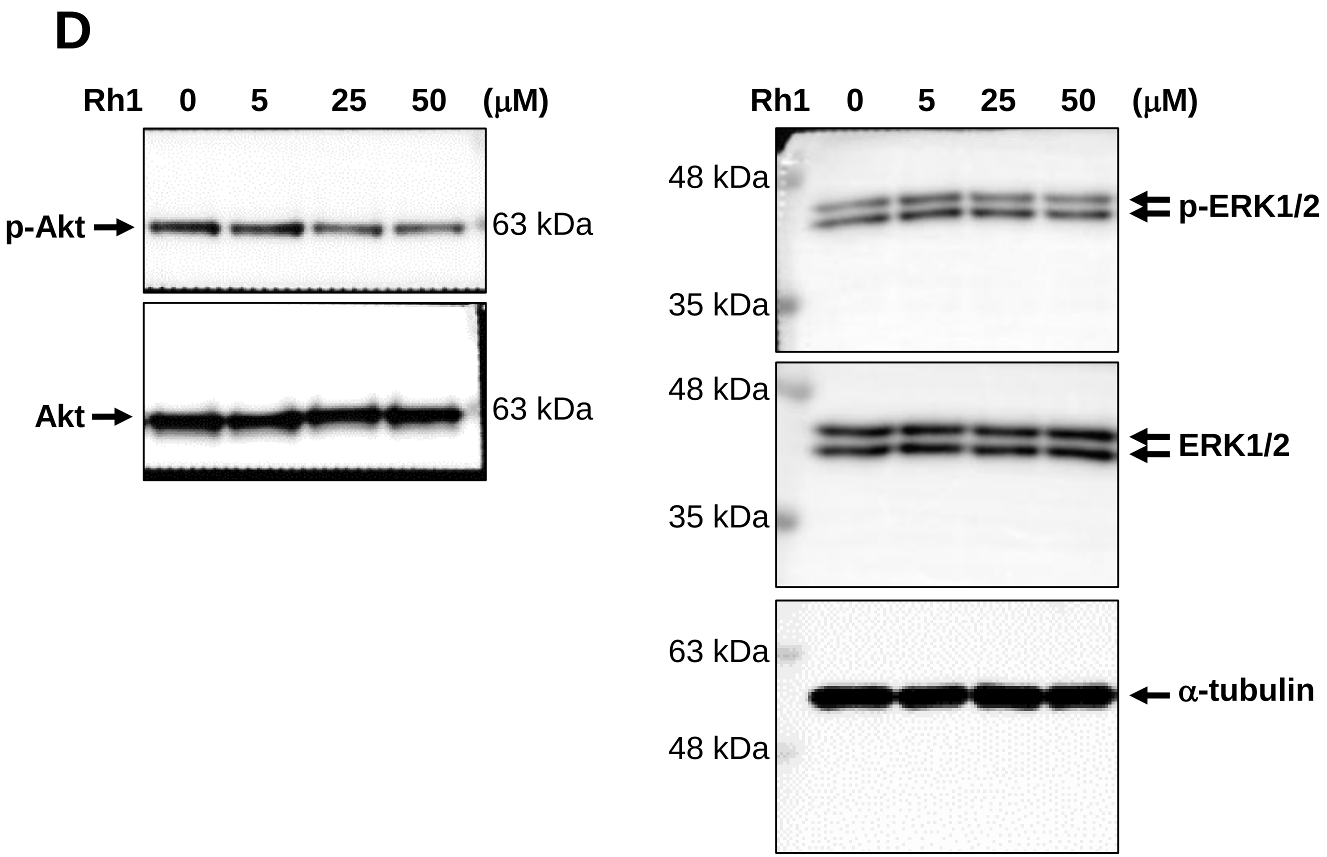
D
Rh1 0 5 25 50 (μM)
p-Akt
63 kDa
Akt
63 kDa
Rh1 0 5 25 50 (μM)
48 kDa
35 kDa
p-ERK1/2
48 kDa
35 kDa
ERK1/2
63 kDa
48 kDa
α-tubulin

Figure 3

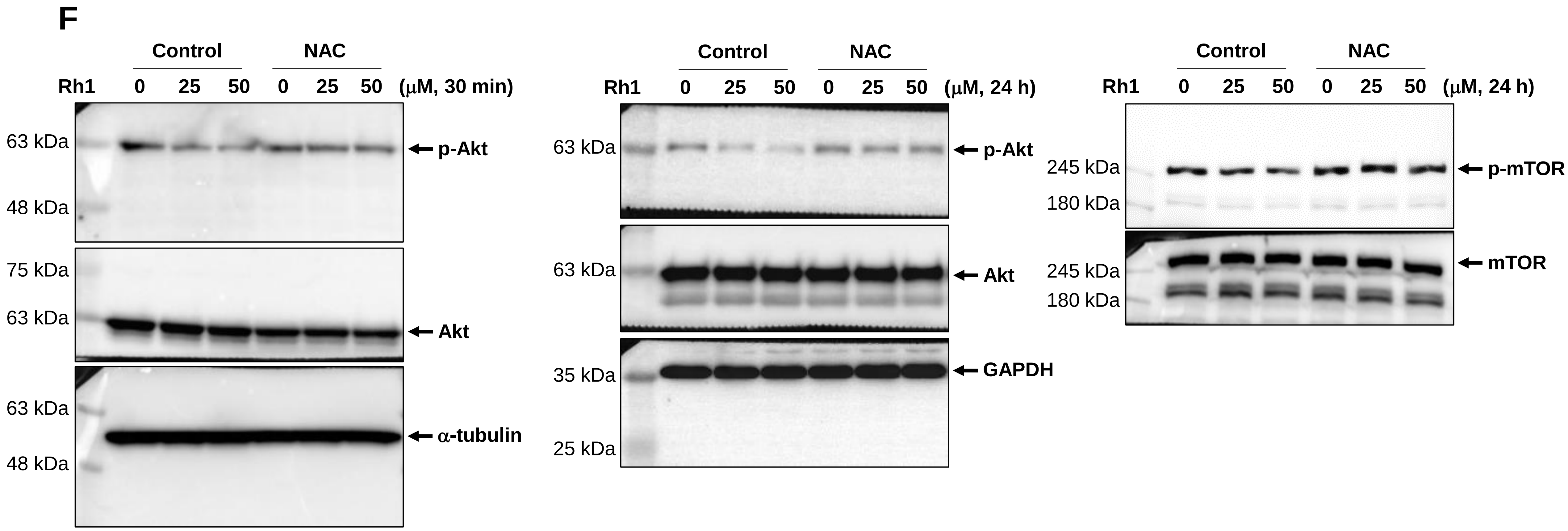
F
Control
NAC
Rh1
0
25
50
0
25
50
(μM, 30 min)
63 kDa
48 kDa
p-Akt
75 kDa
63 kDa
Akt
63 kDa
48 kDa
α-tubulin
Control
NAC
Rh1
0
25
50
0
25
50
(μM, 24 h)
63 kDa
p-Akt
63 kDa
Akt
35 kDa
25 kDa
GAPDH
Control
NAC
Rh1
0
25
50
0
25
50
(μM, 24 h)
245 kDa
180 kDa
p-mTOR
245 kDa
180 kDa
mTOR

Figure 3

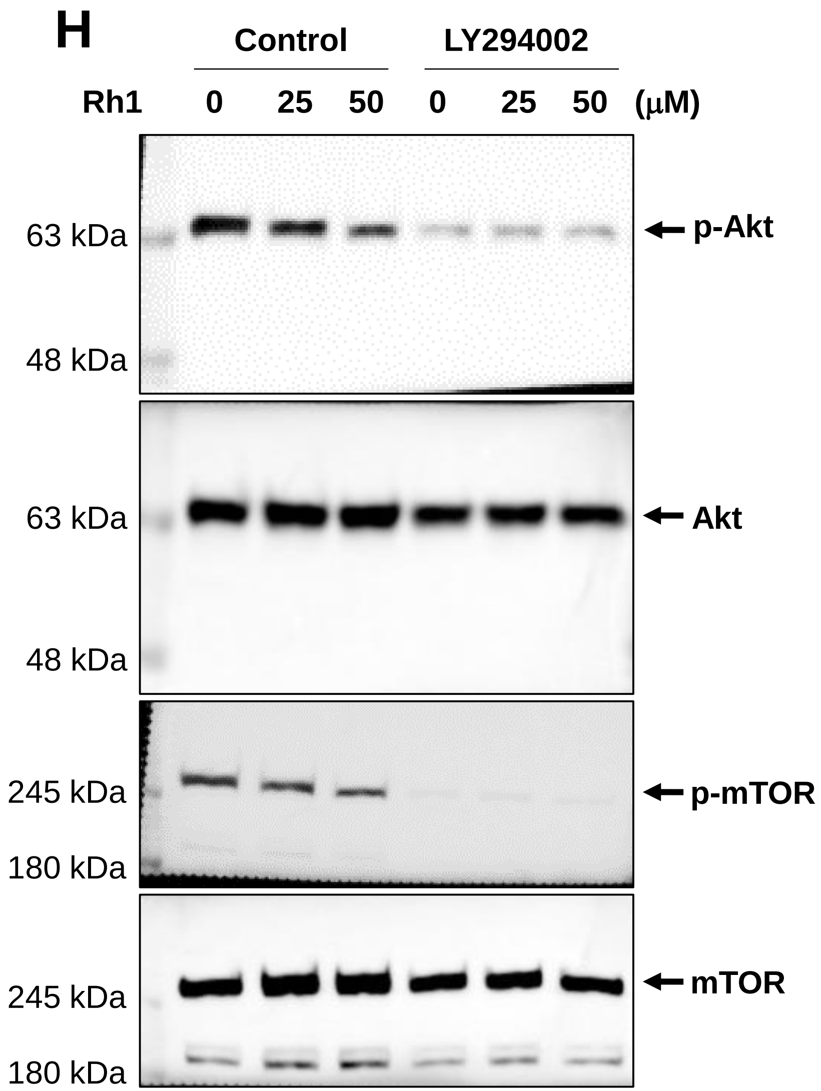

H
Control
LY294002
Rh1
0
25
50
0
25
50
(μM)
63 kDa
48 kDa
p-Akt
63 kDa
48 kDa
Akt
245 kDa
180 kDa
p-mTOR
245 kDa
180 kDa
mTOR

Figure 4

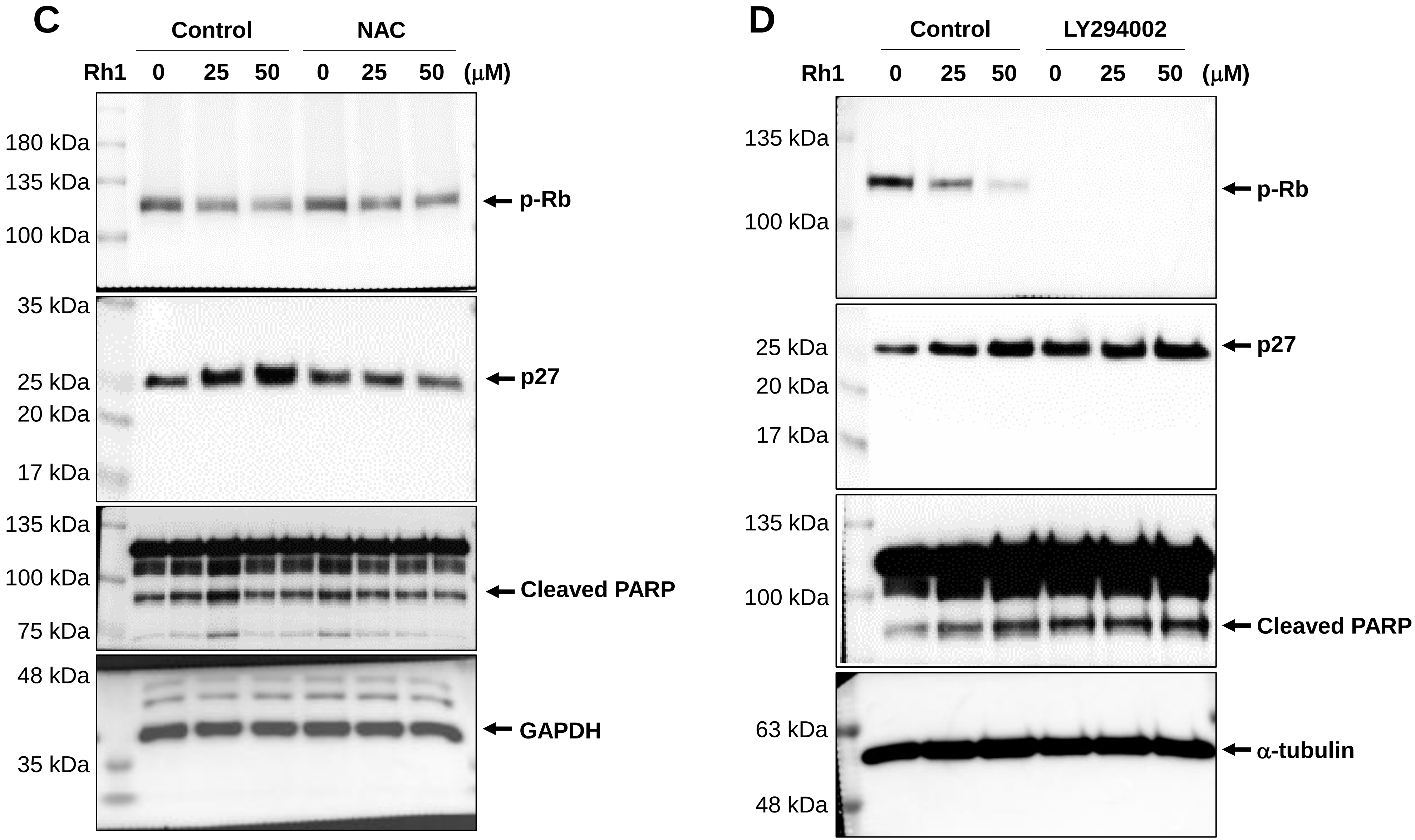
C
Control
NAC
Rh1
0
25
50
0
25
50
(μM)
180 kDa
135 kDa
100 kDa
p-Rb
35 kDa
25 kDa
20 kDa
17 kDa
p27
135 kDa
100 kDa
75 kDa
Cleaved PARP
48 kDa
35 kDa
GAPDH
D
Control
LY294002
Rh1
0
25
50
0
25
50
(μM)
135 kDa
100 kDa
p-Rb
25 kDa
20 kDa
17 kDa
p27
135 kDa
100 kDa
Cleaved PARP
63 kDa
48 kDa
α-tubulin

Figure 5

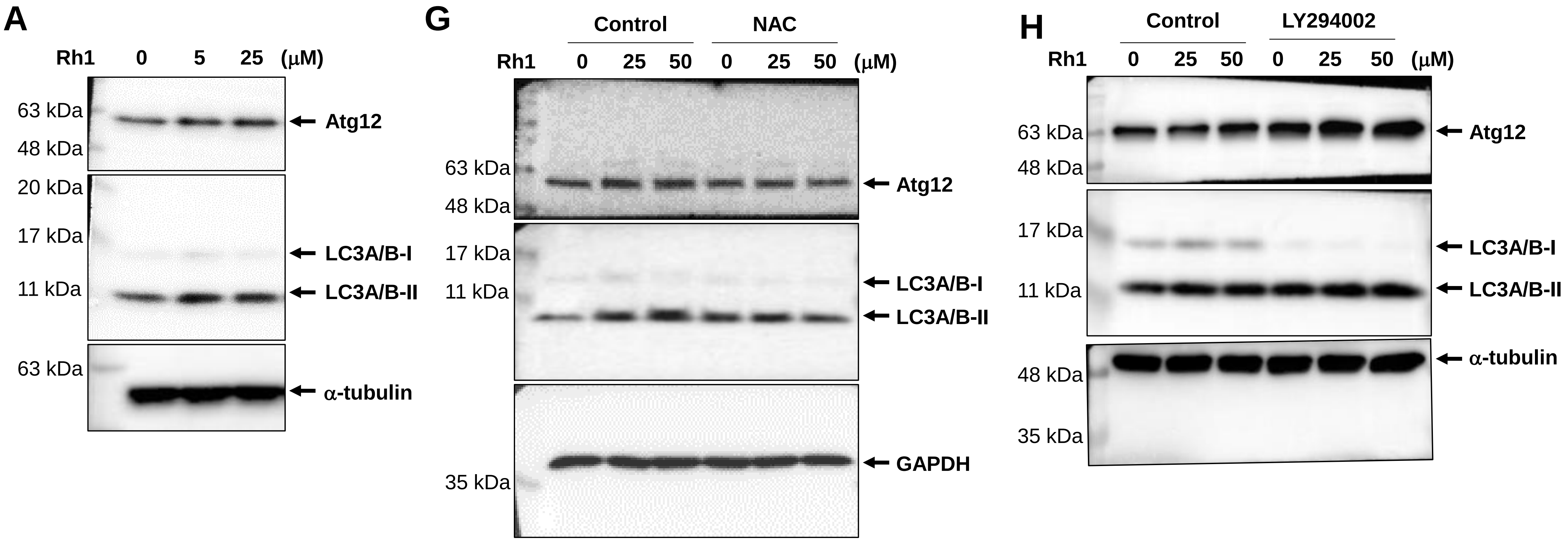
A
Rh1 0 5 25 (μM)
63 kDa
48 kDa
Atg12
20 kDa
17 kDa
11 kDa
LC3A/B-I
LC3A/B-II
63 kDa
α-tubulin
G
Control
NAC
Rh1 0 25 50 0 25 50 (μM)
63 kDa
48 kDa
Atg12
17 kDa
11 kDa
LC3A/B-I
LC3A/B-II
35 kDa
GAPDH
H
Control
LY294002
Rh1 0 25 50 0 25 50 (μM)
63 kDa
48 kDa
Atg12
17 kDa
11 kDa
LC3A/B-I
LC3A/B-II
48 kDa
35 kDa
α-tubulin

Figure 6

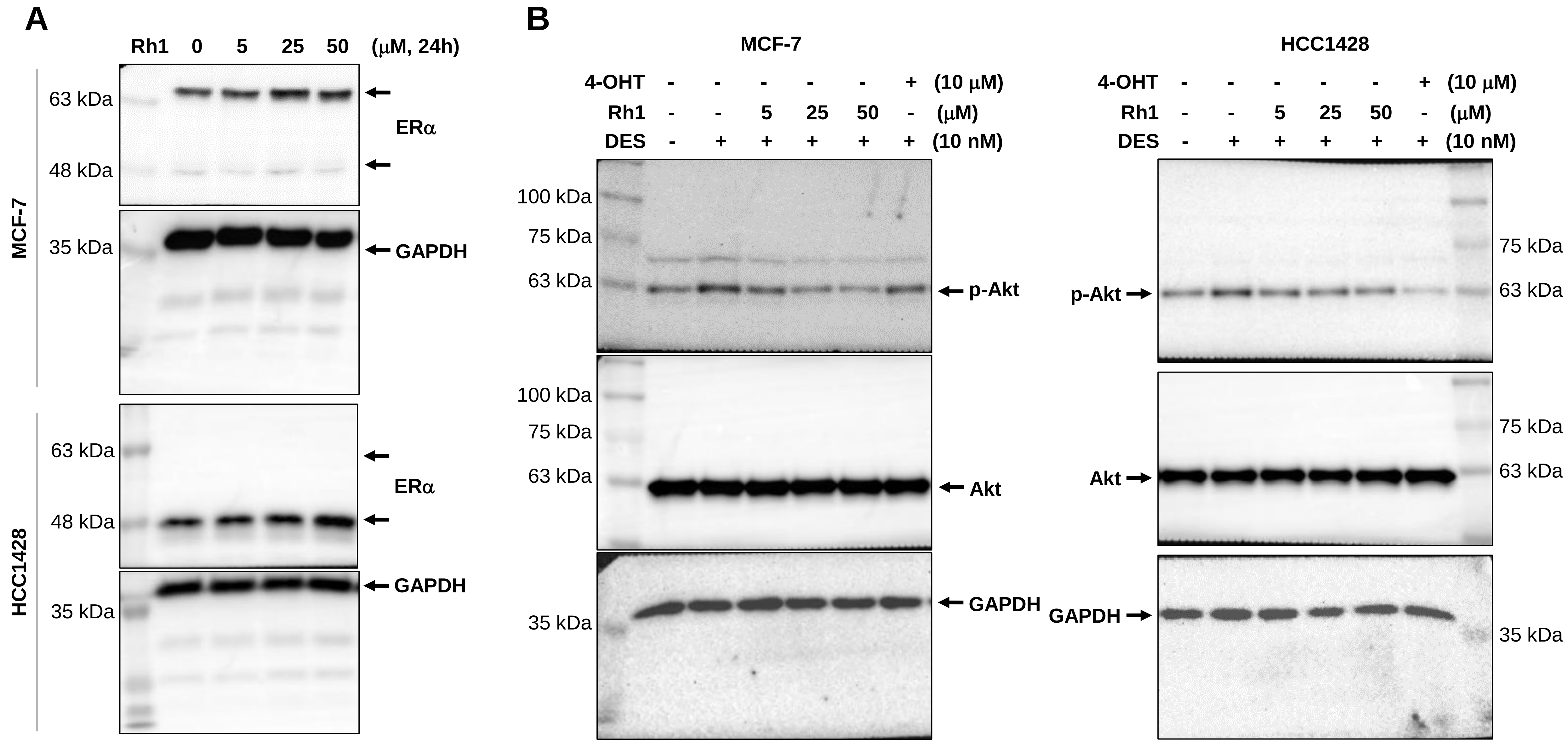
A
Rh1 0 5 25 50 (μM, 24h)
MCF-7
63 kDa
48 kDa
ERα
35 kDa
GAPDH
HCC1428
63 kDa
48 kDa
ERα
35 kDa
GAPDH
B
MCF-7
4-OHT - - - - - + (10 μM)
Rh1 - - 5 25 50 - (μM)
DES - + + + + + (10 nM)
100 kDa
75 kDa
63 kDa
p-Akt
100 kDa
75 kDa
63 kDa
Akt
35 kDa
GAPDH
HCC1428
4-OHT - - - - - + (10 μM)
Rh1 - - 5 25 50 - (μM)
DES - + + + + + (10 nM)
75 kDa
63 kDa
p-Akt
75 kDa
63 kDa
Akt
GAPDH
35 kDa

Figure 6

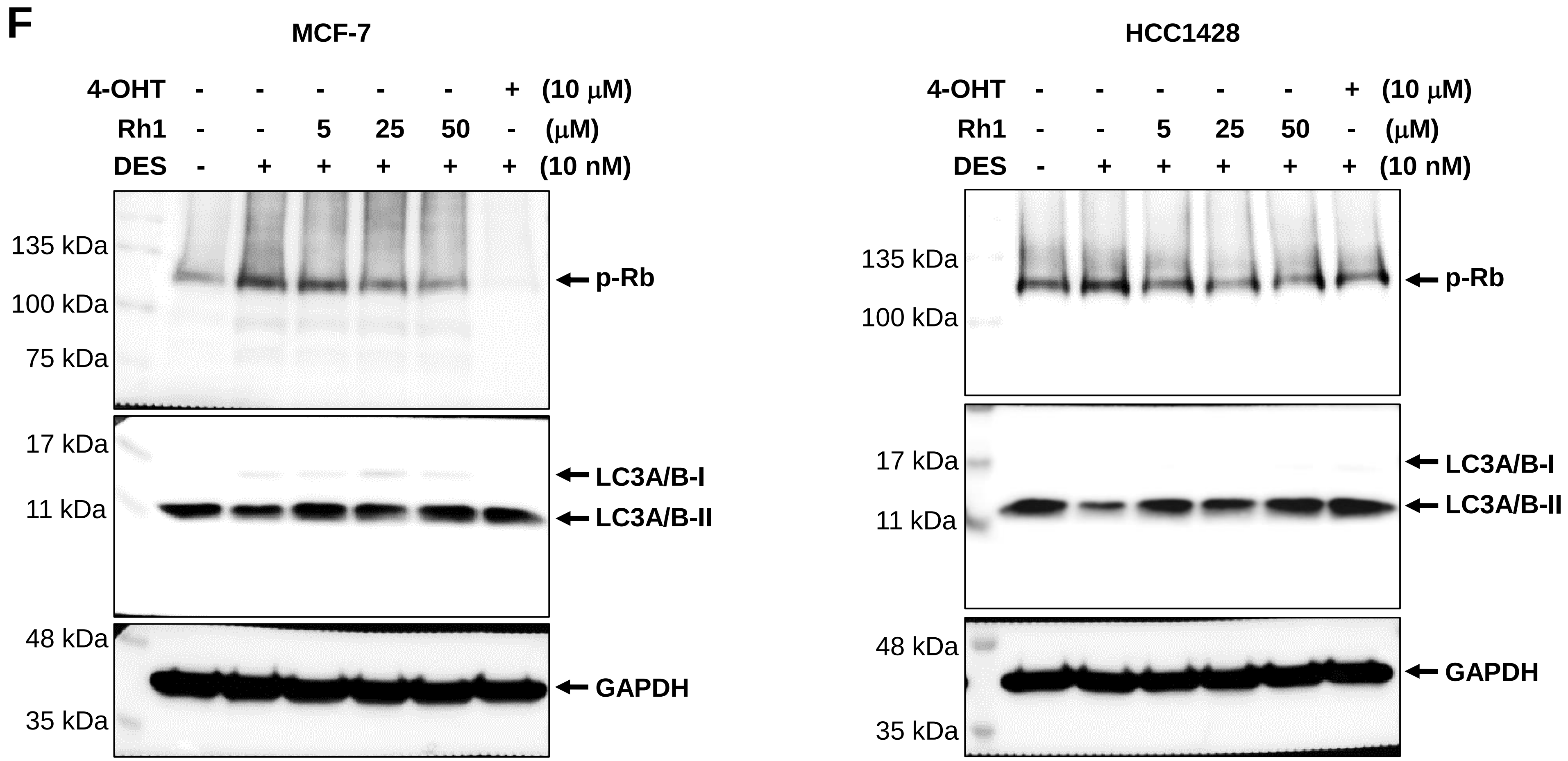
F
MCF-7
4-OHT - - - - - + (10 μM)
Rh1 - - 5 25 50 - (μM)
DES - + + + + + (10 nM)
135 kDa
100 kDa
75 kDa
p-Rb
17 kDa
11 kDa
LC3A/B-I
LC3A/B-II
48 kDa
35 kDa
GAPDH
HCC1428
4-OHT - - - - - + (10 μM)
Rh1 - - 5 25 50 - (μM)
DES - + + + + + (10 nM)
135 kDa
100 kDa
p-Rb
17 kDa
11 kDa
LC3A/B-I
LC3A/B-II
48 kDa
35 kDa
GAPDH

Figure S1

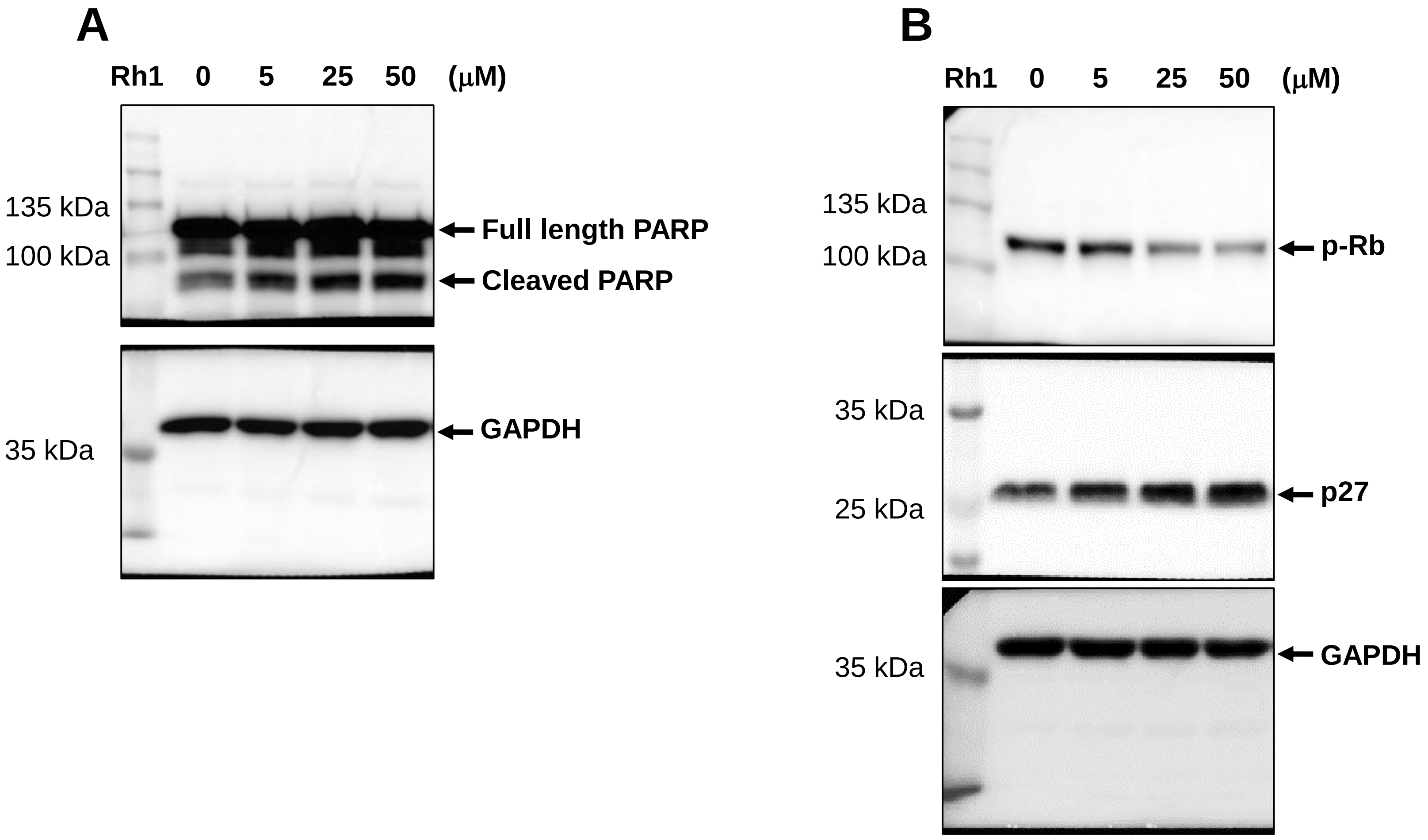
A
Rh1 0 5 25 50 (μM)
135 kDa
100 kDa
Full length PARP
Cleaved PARP
35 kDa
GAPDH
B
Rh1 0 5 25 50 (μM)
135 kDa
100 kDa
p-Rb
35 kDa
25 kDa
p27
35 kDa
GAPDH

Figure S2

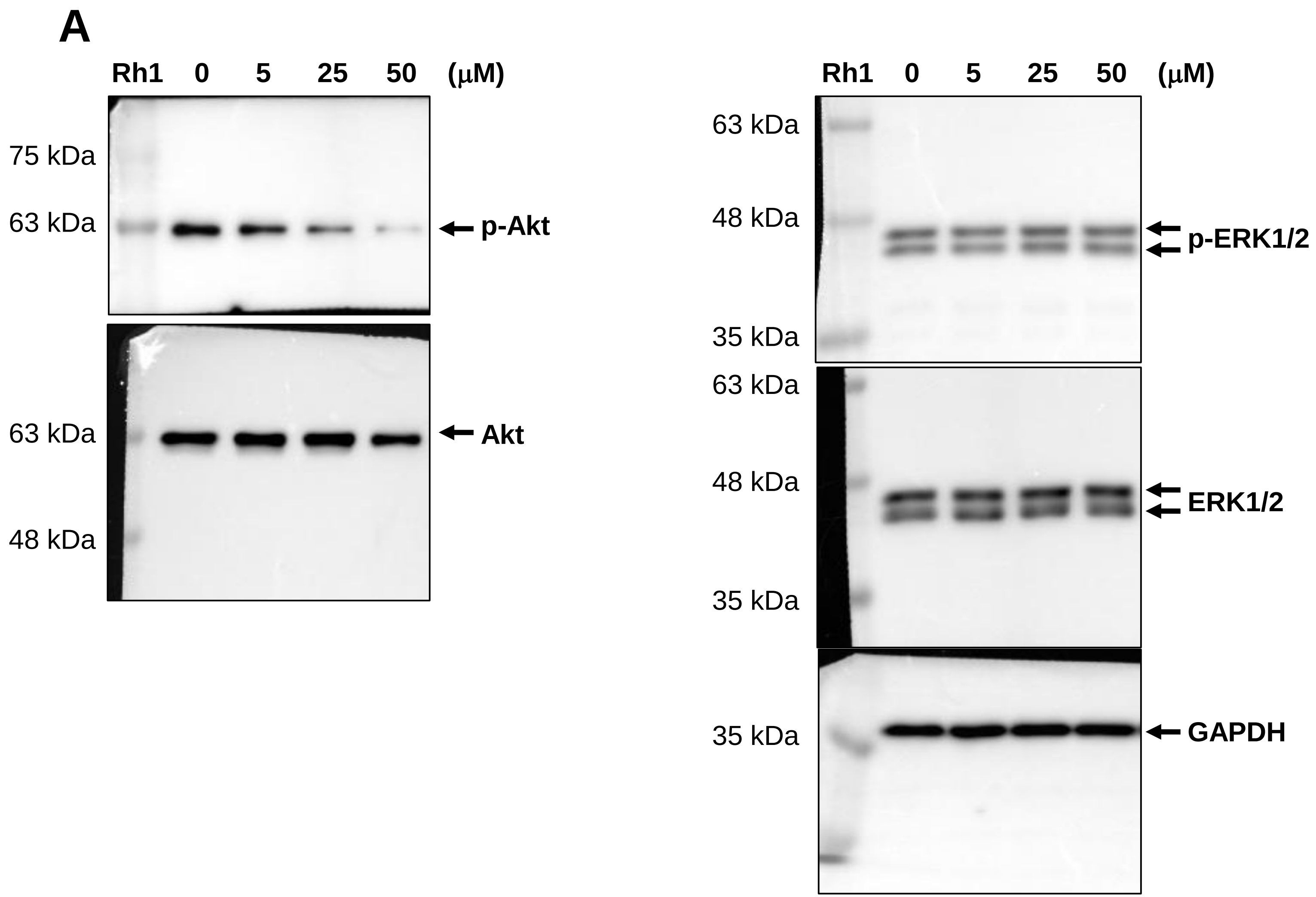
A
Rh1
0
5
25
50
(μM)
75 kDa
63 kDa
p-Akt
63 kDa
Akt
48 kDa
Rh1
0
5
25
50
(μM)
63 kDa
48 kDa
p-ERK1/2
35 kDa
63 kDa
48 kDa
ERK1/2
35 kDa
35 kDa
GAPDH
