# Supplementary material for: Ginsenoside Rh1 Induces MCF-7 Cell Apoptosis and Autophagic Cell Death through ROS-Mediated Akt Signaling
Source: Cancers (Basel). 2021 Apr 15;13(8):1892. doi: 10.3390/cancers13081892 (PMC8071122; doi:10.3390/cancers13081892)
Supplement: Supplementary file 1 [file cancers-13-01892-s001.zip › cancers-1086602-suppl-proof-resubmit.pdf]

Supplementary figures

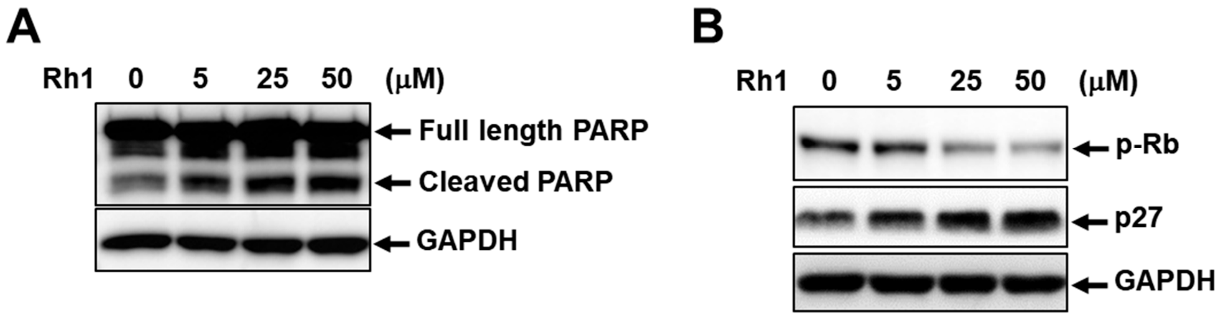

Figure S1. Rh1 induced cell apoptosis and cell cycle arrest in HCC1428 cells. (A, B) The cells were treated with Rh1 (5, 25, and 50 μM) for 24 h. The whole cell lysates then were used to analyze the protein expression as indicated. GAPDH was used as a loading control.

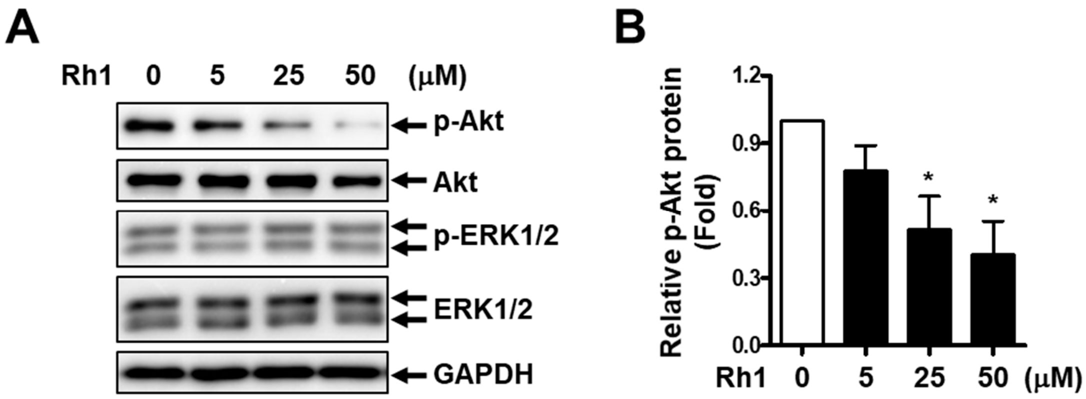

Figure S2. Effects of Rh1 on PI3K/Akt and MAPK pathway in HCC1428 cells. (A, B) HCC1428 cells were treated with Rh1 at various concentrations for 30 min. The whole cell lysates were then used to analyze the kinase activity of ERK1/2 and Akt by western blot (A). The relative quantification of protein levels was analyzed using Image J software (B). Data are expressed as mean ± SD (n=5), *$p < 0.05$ as compared with the control.

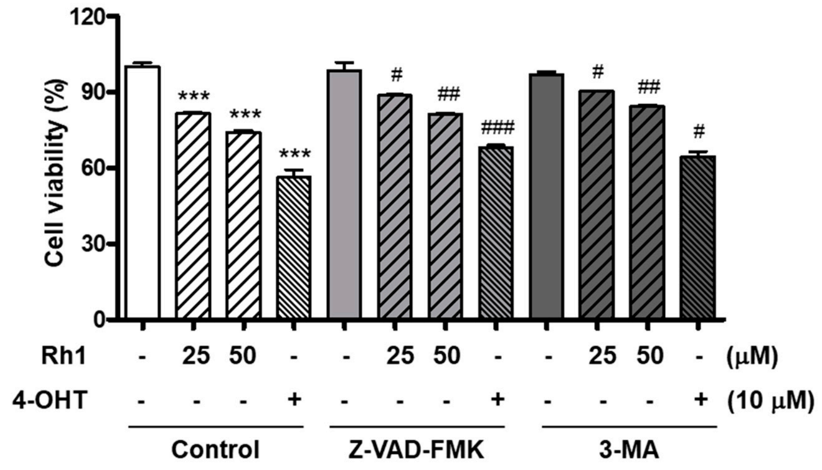

Figure S3. Anti-cancer effect of Rh1 in MCF-7 cells is involved in apoptosis and autophagy. MCF-7 cells were pretreated with Z-VAD-FMK (10 μM) and 3-MA (10 μM) for 1 h, followed by the treatment of Rh1 (25 and 50 μM) or 4-OHT (10 μ) for 24 h. 4-OHT was used as a positive control. Cell viability was evaluated using SRB assay. Data are expressed as means ± SD (*n*=5), ****p* < 0.001 as compared with the control; #*p* < 0.05, ##*p* < 0.01 and ###*p* < 0.001 as compared with Rh1-treated samples. Z-VAD-FMK: caspase inhibitor; 3-methyladenine (3-MA): autophagy inhibitor; 4-OHT: 4-hydroxytamoxifen.

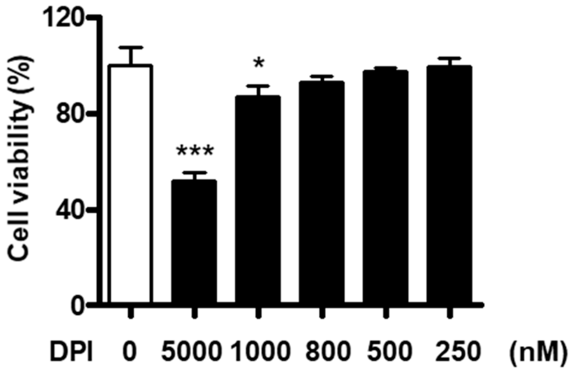

Figure S4. Effect of diphenyleneiodonium (DPI) on MCF-7 cell viability. MCF-7 cells were treated with different concentrations of DPI. MTT assay was then performed to evaluate cell viability. Data are expressed as means ± SD (*n*=5), **p* < 0.05, ****p* < 0.001 as compared with the control.
